# Analysis of the human monocyte-derived macrophage transcriptome and response to lipopolysaccharide provides new insights into genetic aetiology of inflammatory bowel disease

**DOI:** 10.1371/journal.pgen.1006641

**Published:** 2017-03-06

**Authors:** J. Kenneth Baillie, Erik Arner, Carsten Daub, Michiel De Hoon, Masayoshi Itoh, Hideya Kawaji, Timo Lassmann, Piero Carninci, Alistair R. R. Forrest, Yoshihide Hayashizaki, FANTOM Consortium, Geoffrey J. Faulkner, Christine A. Wells, Michael Rehli, Paul Pavli, Kim M. Summers, David A. Hume

**Affiliations:** 1 The Roslin Institute and Royal (Dick) School of Veterinary Studies, University of Edinburgh, Easter Bush, Midlothian, United Kingdom; 2 RIKEN Omics Science Center (OSC), 1-7-22 Suehiro-cho, Tsurumi-ku, Yokohama, Japan; 3 Australian Institute for Bioengineering and Nanotechnology (AIBN), University of Queensland, Brisbane St Lucia, Queensland, Australia; 4 Department of Internal Medicine III, University Hospital Regensburg, F.-J.-Strauss Allee 11, Regensburg, Germany; 5 Medical School, Australian National University, Canberra, ACT, Australia; Icahn School of Medicine at Mount Sinai, UNITED STATES

## Abstract

The FANTOM5 consortium utilised cap analysis of gene expression (CAGE) to provide an unprecedented insight into transcriptional regulation in human cells and tissues. In the current study, we have used CAGE-based transcriptional profiling on an extended dense time course of the response of human monocyte-derived macrophages grown in macrophage colony-stimulating factor (CSF1) to bacterial lipopolysaccharide (LPS). We propose that this system provides a model for the differentiation and adaptation of monocytes entering the intestinal lamina propria. The response to LPS is shown to be a cascade of successive waves of transient gene expression extending over at least 48 hours, with hundreds of positive and negative regulatory loops. Promoter analysis using motif activity response analysis (MARA) identified some of the transcription factors likely to be responsible for the temporal profile of transcriptional activation. Each LPS-inducible locus was associated with multiple inducible enhancers, and in each case, transient eRNA transcription at multiple sites detected by CAGE preceded the appearance of promoter-associated transcripts. LPS-inducible long non-coding RNAs were commonly associated with clusters of inducible enhancers. We used these data to re-examine the hundreds of loci associated with susceptibility to inflammatory bowel disease (IBD) in genome-wide association studies. Loci associated with IBD were strongly and specifically (relative to rheumatoid arthritis and unrelated traits) enriched for promoters that were regulated in monocyte differentiation or activation. Amongst previously-identified IBD susceptibility loci, the vast majority contained at least one promoter that was regulated in CSF1-dependent monocyte-macrophage transitions and/or in response to LPS. On this basis, we concluded that IBD loci are strongly-enriched for monocyte-specific genes, and identified at least 134 additional candidate genes associated with IBD susceptibility from reanalysis of published GWA studies. We propose that dysregulation of monocyte adaptation to the environment of the gastrointestinal mucosa is the key process leading to inflammatory bowel disease.

## Author summary

Macrophages are immune cells that form the first line of defense against pathogens, but also mediate tissue damage in inflammatory disease. Macrophages initiate inflammation by recognising and responding to components of bacterial cells. Macrophages of the wall of the gut are constantly replenished from the blood. Upon entering the intestine, newly-arrived cells modulate their response to stimuli derived from the bacteria in the wall of the gut. This process fails in chronic inflammatory bowel diseases (IBD). Both the major forms of IBD, Crohn’s disease and ulcerative colitis, run in families. The inheritance is complex, involving more than 200 different regions of the genome. We hypothesised that the genetic risk of IBD is associated specifically with altered regulation of genes that control the development of macrophages. In this study, we used the comprehensive transcriptome dataset produced by the FANTOM5 consortium to identify the sets of promoters and enhancers that are involved in adaptation of macrophages to the gut wall, their response to bacterial stimuli, and how their functions are integrated. A reanalysis of published genome-wide association data based upon regulated genes in monocytes as candidates strongly supports the view that susceptibility to IBD arises from a primary defect in macrophage differentiation.

## Introduction

Inflammatory bowel disease (IBD) comprises a group of complex syndromes that arise from a dysfunctional interaction between the microbiota of the intestinal lumen and the immune system [1]. Loci associated with the heritability of susceptibility to IBD are shared in some measure with other chronic inflammatory diseases [2,3]. Extensive genome-wide association studies (GWAS) have identified more than 200 risk loci for IBD, with significant overlaps between the two major forms, Crohn’s disease and ulcerative colitis [4–6]. The analysis of candidate genes within susceptibility loci based upon apparently shared biological function has emphasised three major pathways: the activation of Th17 T cells, autophagy, and the response to mycobacteria [7,8]. The emphasis on T cell activation derives in part from a clear association of susceptibility with certain MHC haplotypes, and the observed activation of T cells in inflamed mucosa [9]. An alternative view is that IBD is primarily initiated by functional dysregulation in cells of the macrophage lineage [10,11] and prioritisation of candidates on that basis may be informative [12].

By contrast to other tissue macrophage populations which have a significant capacity for self-renewal, lamina propria macrophages of the gut are renewed continuously from the circulating monocyte pool [13,14]. The proliferation and differentiation of the monocyte-macrophage lineage is controlled by the growth factor, macrophage colony-stimulating factor (CSF1), which signals through a tyrosine kinase receptor, CSF1R [15–17]. The replenishment of the resident cells of the lamina propria requires the continuous exposure to CSF1 and lamina propria macrophages are rapidly depleted in mice treated with a blocking anti-CSF1R antibody [18,19]. Human macrophages are commonly generated by cultivation of isolated CD14^+^ monocytes for 4–5 days in CSF1 [20,21]. Several groups have published detailed transcriptomic analysis of the differentiation of monocytes in CSF1 [20–23]. Based upon the known biology (derivation from monocytes, dependence upon CSF1), we suggest that the monocyte-derived macrophage actually approximates an *in vitro* model of the differentiation of intestinal macrophages from incoming monocytes.

Alongside CSF1, monocytes entering the lamina propria of the lower GI tract are immediately exposed to microbial products of which the archetype is lipopolysaccharide (LPS) from gram-negative bacteria. Monocytes must rapidly down-modulate their response to bacteria in the lumen, to avoid initiating an inflammatory response in the gut wall [18]. In response to LPS, macrophages initiate a complex feed-forward and feed-back cascade of induction and repression of transcription factors and autocrine regulators [24,25] leading to a new steady state. The response of mouse macrophages to LPS has been studied in detail at many levels from the mechanisms of signaling [26,27] through transcriptional networks [28–30] to the underlying alterations in chromatin structure [31–33]. Dense time course data are required to enable inference of the sequence of transcriptional events in this response.

The FANTOM consortium established tag sequencing of genome-scale 5’-RACE (CAGE) as an expression profiling tool and used the approach to create a comprehensive human promoter-based expression atlas [34]. Deep sequencing of CAGE libraries also detected the bidirectional transcripts (eRNAs) derived from active enhancers enabling genome-wide quantification of enhancer activity [35]. CAGE-based delineation of transcription start sites was used in the comparison of the promoters of LPS-inducible genes in mouse and human macrophages [21] as well as a recent detailed analyses of monocyte subsets [36]. Extensive analysis of cells undergoing state change showed a clear temporal pattern in induction of expression; enhancers are expressed first, then transcription factor genes, and finally the genes they regulate [37].

In the current study, we analysed CAGE data generated within FANTOM5 to dissect the transcriptional changes that occur in human macrophages grown in CSF1, and their subsequent response to LPS, as a model for events that occur when monocytes adapt to the gut environment. We use these data, combined with extensive data on the gene expression of blood monocytes generated by the FANTOM5 consortium, to reassess the known candidate intervals associated with IBD. Our analysis supports the hypothesis that IBD is primarily a macrophage-initiated pathology and provides the basis for identification of alternative candidate genes within many IBD susceptibility loci that have been identified by genome-wide analysis (GWA).

## Results

### Description of the datasets

The aim of this study was to examine the hypothesis that dysregulation of intestinal macrophage responses to bacterial antigens is an important component of susceptibility to IBD. Initially, as part of the FANTOM5 project, we produced a detailed quantitative analysis of promoter utilization and gene expression across a dense time course of the LPS response of human monocyte-derived macrophages (MDM) from three separate donors. The time course focused on very early events (at 15 minute intervals from initiation of the response), as well as the later change in differentiation state up to 48 hours after stimulation. For comparison, the FANTOM5 dataset contains three additional unstimulated MDM samples from different donors, obtained commercially, three separate CD14^+^ monocyte populations, each in triplicate, and cultured monocytes stimulated for 2 hours with a range of agonists including gamma interferon (IFNγ) LPS and live salmonella. The monocytes differed in their method of isolation. One set, also purchased commercially, was clearly “activated” and expressed many inflammatory cytokines. Another set was divided into the three known monocyte sub-populations based upon relative expression of CD14 and CD16, as described in detail elsewhere [36,38]. The entire FANTOM5 dataset, including an extensive mouse CAGE-based transcriptomic dataset is available on the ZENBU browser (see below). All references in the text to the level of expression, or the temporal expression profiles of individual genes within the text can be confirmed by accessing this browser and entering the gene name.

### Transcriptional landscape of the macrophage response to LPS

**Fig 1:**
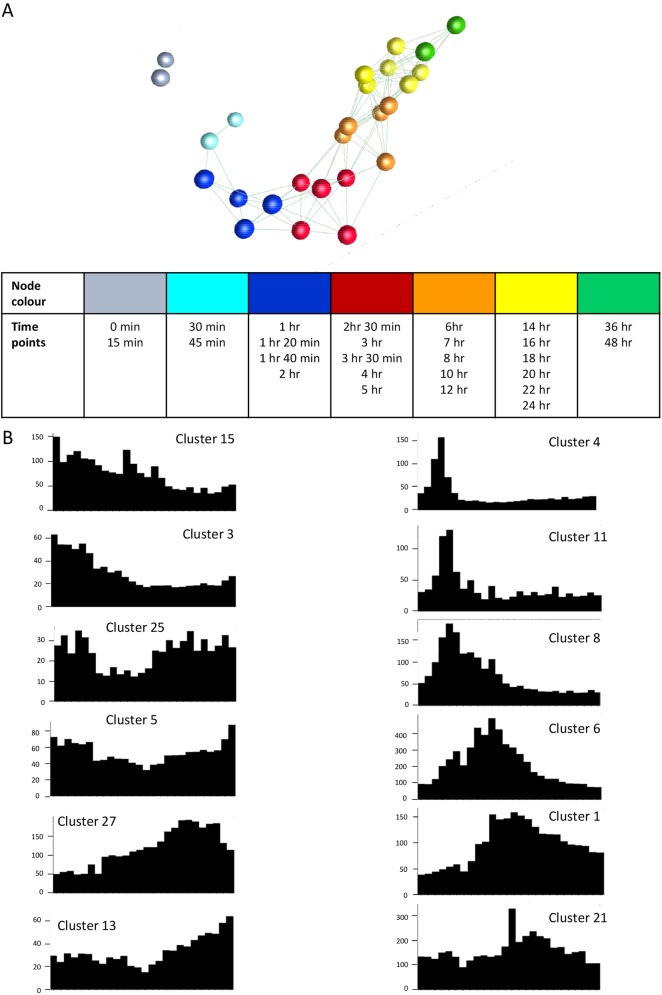
Transcription factor gene expression during stimulation of human monocyte derived macrophages by LPS. *A*. *Sample-based network of transcription factor gene expression*. Gene-based expression levels for all of the annotated transcription factors in the human genome [34] were extracted from the FANTOM5 data and averaged. Only transcription factor genes where at least one value (averaged across three replicates) was ≥ 20 TPM were included in the analysis. The results were entered into BioLayout *Express*^3D^ to create a sample-to-sample network layout where the nodes (spheres) represent a single time point after stimulation with LPS (averaged over three replicates). Edges (lines between nodes) represent sample-to-sample correlations in overall expression pattern of transcription factor genes of R ≥ 0.90. The time points are group and color-coded as indicated, showing the clear progressive change in the transcriptional profile with time. *B*. *Representative expression profiles for genes expressed at the different time points*. Clusters of co-regulated transcription factor genes were identified based upon gene-to-gene network analysis with BioLayout *Express*^3D^ (R ≥ 0.75; MCL inflation value 2.2) as described in Methods. The X axis shows the time points (in the order 0 min, 15 min, 30 min, 45 min, 1 hour, 1 hour 20 min, 1 hour 40 min, 2 hours, 2 hours 30min, 3 hours, 3 hours 30 min, 4 hours, 5 hours, 6 hours, 7 hours, 8 hours, 10 hours, 12 hours, 14 hours, 16 hours, 18 hours, 20 hours, 22 hours, 24 hours, 36 hours, 48 hours). Y axis shows the mean TPM value for the set of genes with that profile (averaged across three replicates). Note that each cluster has a characteristic temporal profile. The complete set of clusters and the names of the transcription factor genes within them is provided in S2 Table.

Previous studies of mouse macrophage response to LPS revealed sequential induction and repression of numerous transcription factors [30]. To gain an overview of the transcription regulatory cascade of human macrophage response to LPS, we first utilized the data visualisation tool Biolayout Express^3D^ [39] to generate a pairwise correlation matrix based upon aggregated expression of the promoter activity of annotated transcription factors identified previously [34]. The sample-to-sample graph in Fig 1, based solely upon the set of transcription factors, clearly shows that the response to LPS can be visualized as a progressive and profound change in transcription factor milieu. Gene-to-gene analysis showed that this involves at least 200 distinct transcription factors. Fig 1 shows the averaged expression profiles of the transcription factor genes within selected clusters, identified by the gene-to-gene analysis, emphasizing that the transcription factor genes can be classified based upon the peak time of induction and whether or not induction was sustained. The detailed list of the transcription factor genes within all of the co-regulated clusters is provided in S1 Table. Many of these transcription factors are discussed below.

CAGE is essentially genome-scale 5’ RACE. Unlike microarrays, CAGE tag sequencing resolves the separate and independent utilization of alternative promoters for the same gene, and is significantly more sensitive across a large dynamic range [34]. For example, the CAGE data reveal that *SERPINA1* has at least 4 promoters. Macrophages and other myeloid cells profiles by the FANTOM5 consortium selectively utilize individual promoters p2, p3 and p4 of the *SERPINA1* gene, whereas p1 is liver-specific. Because it sequences only the 5’ ends, CAGE does not require normalisation for the length of the transcript, which is necessary for RNA sequencing (RNAseq) data, and is considerably more cost-effective than RNAseq. Extensive validation of the CAGE methodology has been published elsewhere, showing the strong correlation of CAGE tag peaks with tissue-specific chromatin marks and DNase hypersensitive sites, and the precise colocation of the 5’ ends of the CAGE tags with the 5’ ends of full length transcripts and promoter-associated motifs such as the TATA box [34–36]

**Fig 2:**
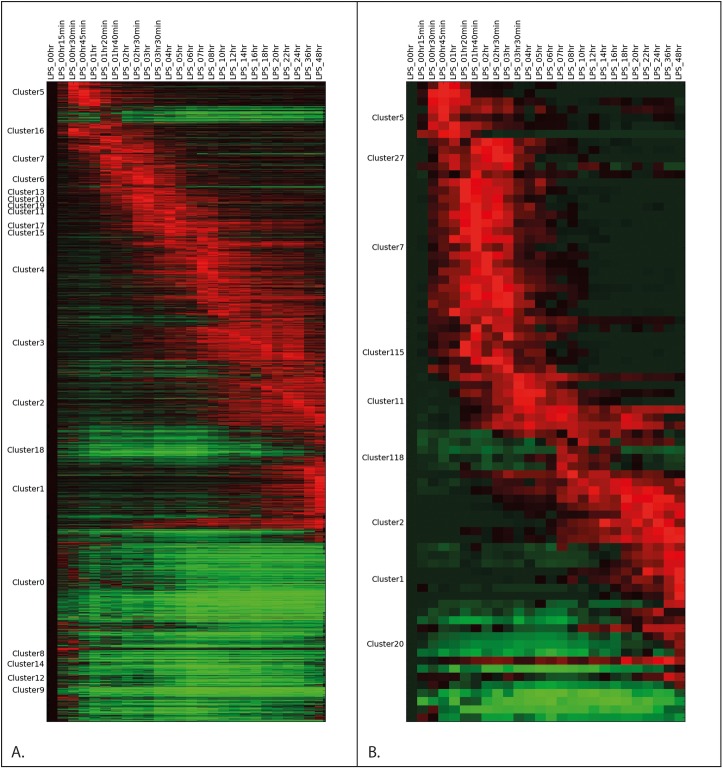
Transcript expression in human macrophages exposed to LPS. Heatmaps of transcript expression in human macrophages exposed to LPS depicting change from baseline, normalized to the maximum expression of each transcript. Red = increase from baseline, green = decrease from baseline. Only nodes included in a coexpression cluster are shown here. (A) All clustered transcripts in LPS-treated human monocyte-derived macrophages. Clusters containing more than 50 transcripts are labeled. (B) Transcripts contained within the clusters shown in (A) that initiate near a putative Crohn’s disease-associated SNP (p<10e-6, window size to transcription start site = 2000 bases).

We created a pairwise correlation network of individual promoters (defined as cluster of transcription start sites, or CTSS) of all expressed genes (Fig 2). Because most genes have several alternative promoters, the same gene can appear in multiple coexpression clusters. The gene-to-gene (or more correctly, promoter-to-promoter) clusters, with graphical displays of their average expression profiles can be accessed at http://coexpression.roslin.ed.ac.uk/lps, and the gene names within selected clusters discussed in the text are also provided in S2 Table, together with a summary of the pattern of expression. In keeping with the regulation of transcription factors in Fig 1, the analysis revealed clusters of coexpressed promoters that have peak activity at different times during the time course. The complex pattern is shown in a network graph produced with the software Biolayout Express^3D^ in S1 Fig. The network graph groups sets of transcripts with related temporal profiles. The positions of clusters on the graph progressively diverge from each other as sets of transcripts are induced or repressed compared to the initial state. The clusters characterized by either early or late expression profiles are the most similar to the unstimulated state. So, the network graph forms a circle. The data are presented in more conventional heat map form in Fig 2. The heat map highlights the cascade of transient regulation of gene expression with time in response to LPS, and the fact that no new steady state is reached even after 48 hours. Within the first 15–30 minutes, there was a set of genes that was rapidly induced and another set that was repressed. Thereafter, there were waves of transient induction of thousands of promoters, each of which peaks at different time points, ranging from 30–45 mins until 36–48 hours. In addition to those clusters that were induced transiently, there were others that were induced in a sustained manner, so that at the 48 hour time point, the transcriptional state of the stimulated cells was still changing.

These coexpressed promoter clusters have been numbered in order based upon the number of annotated genes they contain. The largest clusters, are slowly-repressed (Cluster 0) or slowly-induced (Clusters 1, 2, 3, 4) peaking at different times late in the time course. Cluster 0 contains many known cell cycle genes, and presumably reflects in part the cessation of cell division in a subset of cells growing in CSF1. Clusters 1–4 are all enriched for innate immune genes. Together, these slow-response clusters paint a picture of the progressive differentiation of the cells, which parallels the progressive change in transcription factor milieu shown in Fig 1.

### Induction of immediate early genes by LPS

Immediate early genes are a set of transcriptional regulators induced in many different cell types as they transition from one cellular state to another. Cluster 5 (S1 Fig, Fig 2, S2 Table) contains promoters with detectable increased expression within 15 mins, peaking at 45 mins, and declining rapidly thereafter. This cluster of the earliest inducible promoters contains genes encoding many immediate early transcription factors (*EGR1*, *EGR2*, *EGR3*, *FOS*, *FOSB*, *JUN*) [37]. One of the most inducible, and the most highly-expressed, transcriptional regulators in the LPS response is *NFKBIZ*, which encodes IκB-ζ. *NFKBIZ* has two promoters, both of which were LPS-inducible. Induction of IκB-ζ is likely to be critical for subsequent events in the transcriptional cascade [40]. In mouse macrophages, it interacts with AKIRIN2, to bridge NFκB and the chromatin remodeling SWI/SNF complex on proinflammatory genes including *IL6* and *IL12B* in macrophages [41]. The early-inducible transcription factors included genes encoding factors more commonly associated with lymphocyte functions; *NFIL3*, *IKZF1*, *KLF2* and *PRDM1*.

Two smaller clusters, 30 and 42 (S2 Table), were induced marginally later, peaking around 1hr 20 minutes. This group contained *IFNB1*, a target of NFκB signaling which is known to act in an autocrine manner to induce the interferon target genes (see below). Other inducible feed-forward activators include the gene encoding the adaptor protein TRAF1, which interacts directly with signaling molecule MAP3K5 (also known as ASK1) which in turn is involved in LPS signaling [42] and the gene encoding EGR4, which along with EGR3, can interact with NFκB and probably contribute to activation of NFκB target genes [43].

### The inflammatory cytokine clusters

Genes for the well-known inflammatory cytokines (*IL1B*, *TNF*, *IL6*, *IL10*) and chemokines that are the hallmark of the response to LPS form parts of Clusters 6 and 7 (S2 Table). A previously unreported member of this group was *TNFSF9*, or *CD137L*, which was massively-induced by LPS and has been proposed as a growth and differentiation factor for myeloid cells [44]. Expression of the proinflammatory genes differed from immediate early response genes in terms of the duration of the expression. For example, *TNF* mRNA detected by CAGE was induced 10-fold within 15 mins, but was still somewhat elevated after 5–6 hours. The small MAF transcription factor gene, *MAFF*, was strongly induced alongside the inflammatory cytokines. The other members of this family (*MAFG*, *MAFK*) were also induced by LPS, but later in the time course. The small MAF transcription factors form heterodimers with many other transcription factors, notably the NFE2 family (*NFE2*, *NFE2L1*, *NFE2L2*, *NFE2L3* genes) [45] all of which are induced by LPS later in the time course. Hence, induction of *MAFF* is likely to be part of a feed forward cascade.

### Interferon-response genes

Interferons (IFNs) and their targets are a key part of innate immune defence mechanisms, and chronic over-expression of IFN target genes is a feature of many inflammatory diseases [46–49]. The immediate early induction of *IFNB1* in response to LPS initiates an autocrine loop that leads in turn to downstream induction of the targets of IFN signaling [27,33,47,50]. Many known IFN targets were induced by LPS and formed parts of coexpressed clusters, but distinct clusters containing these genes followed distinct temporal profiles. Based upon a search of the Interferome database [51], a total of 123 genes within Clusters 2 and 3 (the large late-response clusters noted above) have previously been shown to respond directly to IFN signaling in human monocytes or monocyte-derived macrophages. [51]. Several smaller clusters of known IFN-response genes were induced significantly earlier. One, Cluster 10 (S2 Table), peaked at 2–3 hours, and included the *IFIT2*, *3* and *5* genes that derive from a single genomic region as well as genes for known virus/IFN-induced chemokines, CCL3 and 4 and CXCL10 [52]. Other clusters showing an early peak in expression contained genes for the known inducible feedback regulators of IFN-induced JAK-STAT signaling, *SOCS1* and *SOCS3* [53,54]. Cluster 11 peaked around 1 hour later than Cluster 10, and Cluster 17 peaked slightly later again, and declined more slowly, similar to Clusters 33 and 49. [53,54].

The diversity of responses of IFN-inducible genes probably reflects the complex regulation of the transcription factors that bind to interferon-response elements (IRFs). The canonical MyD88-independent, TRIF/TRAM (TICAM1/2)-dependent pathway of IFN regulation determined from studies of mice involves the interactions of TRAF3 and the kinase TBK1 to phosphorylate IRF3 [55]. However, this pathway is not conserved in humans [21]. In the human MDM, *IRF3* mRNA was almost undetectable, and both *TRAF3* and *TBK1* were very low. Genes encoding the adaptor molecule TICAM1, and the downstream RIK kinases (RIPK1, RIPK2) were induced by LPS, peaking after 2–3 hours with Cluster 13.

The most-rapidly-induced IRF family member in the LPS-stimulated human MDM was *IRF1*. This observation is consistent with recent direct evidence of LPS-induced IRF1 binding to promoters and enhancers of inflammatory cytokine genes (*TNF*, *IL6*, *IL12B*) in human MDM, and its role in the priming response to IFNγ [56]. *IRF1* mRNA was induced detectably within 15 mins, peaked around 2 hours and was sustained at greatly elevated levels for the remainder of the time course. *IRF4* was part of cluster 17, maximally induced at 3–4 hours and declining thereafter. *IRF7* was induced marginally later and remained elevated throughout, *IRF8* was induced transiently, peaking at 3 hours and gone by 12 hours, *IRF2* and *IRF9* were induced in late response clusters. Other components of the IFN signaling pathway, notably *STAT1*, *STAT2* and *STAT4*, and *JAK2* and *JAK3* were also induced transiently. It is likely that each of these factors contributes specifically to transcription regulation of the clusters in which they are coexpressed possibly by interacting with other induced transcription factors. For example, IRF8 is known to bind uniquely to a specific subset of IRF recognition sequences [29].

### Inducible autocrine regulatory loops in the LPS response

Members of the FANTOM Consortium have produced a cellular interactome linking coexpressed ligands and receptors [57]. The progressive response of MDM to LPS emphasized in Figs 1 and 2 and S1 and S2 Tables is based upon part upon numerous autocrine loops in which induction of ligands is followed by induction of the receptors. The most obvious is the response to endogenous IFNB1; the genes for receptors for type 1 interferon, *IFNAR1* and *IFNAR2*, were expressed in MDM and further induced starting around 5–6 hours. TNF has also been shown to initiate an autocrine loop in mouse macrophages responding to LPS, so that induction of some late response genes is ablated in *TNF* knockout mice [58]. Both TNF receptor genes *TNFR1* (*TNFRSF1A*) and *TNFR2* (*TNFRSF1B*) were induced later in the response to LPS. Aside from *TNF*, five other members of the TNF superfamily, *TNFSF15*, *TNFSF8* (encoding CD30L), *TNFSF10* (TRAIL), *TNFSF14* (LIGHT) and *TNFSF9* (CD137L) were each induced ahead of induction of their respective receptors *TNFRSF6B*, *TNFRSF8*, *TNFRSF10A* and *10B* and *TNFRSF9* (CD137). The rapid induction of prostanoid synthesis is a hallmark of LPS signaling, associated with the early induction of *PTGS2* (COX2)[26]. PGE receptors (*PTGER3*,*4*) and the prostacyclin receptor (*PTGIR*) were strongly induced at later time points in the response. The gene for the vitamin D3-generating enzyme, *CYP27B1*, was strongly-induced by LPS in MDM and the vitamin D3 receptor gene (*VDR*) was induced later in the response to LPS.

Another set of potential autocrine loops was generated through the induction by LPS of *CSF1*, *GMCSF* (*CSF2*) and *GCSF* (*CSF3*), commencing around 2–3 hours after LPS treatment. Their receptors, *CSF1R* and *CSF3R*, were expressed constitutively and *CSF2RB* was strongly-induced by LPS around 4–5 hours. The cytokine *IL15* was induced substantially in MDM by 2 hours, whilst *IL15RA* was induced from undetectable levels starting at 2–3 hours. Genes for both IL27 subunits (*IL27* and *EBI3*) were strongly induced, and MDM expressed *IL27RA* constitutively. Finally, many of the inducible CC chemokines probably produce autocrine signals through CC receptors; some were expressed constitutively, and some (e.g. *CCR1*, *CCRL2*, *CCR5*, *CCR7*) were themselves LPS-inducible with distinct individual time courses.

### Inducible feedback inhibition of the LPS response

One striking feature of the response to LPS is the transience of each successive wave of inducible gene expression (S1 Fig, Fig 2). Each wave probably contains the seeds of its own destruction, inducible repressors that promote decay of the signal and degradation of the induced transcripts and proteins. For example, the set of induced autocrine loops elicited by LPS includes progressive accumulation of the repressive cytokine IL10, alongside its receptor, IL10RB. The LPS time course data supports many known and suggests several novel feedback loops. Amongst the earliest inducible transcription factors in Cluster 5, *PRDM1* (also known as *BLIMP1*) was the most highly-expressed. In mice, the PRDM1 protein has been attributed functions as a transcriptional repressor of cytokine induction [59]. *IKZF1* (ikaros) has also been attributed roles in feedback repression of LPS signaling in mice [60] and was associated with a trans-acting expression quantitative trait locus (eQTL) in a large survey of gene expression in human peripheral blood leukocytes [61]. NR4A1 (encoded by *NR4A1*) has also been implicated as a transcriptional repressor of NFκB signaling in mouse macrophages [62]. Alongside these inducible feedback repressors of transcription, Cluster 5 also contains promoters for feedback regulators of MAP kinase signaling, *DUSP1* and *DUSP2* [63].

All of the inhibitors of NFκB (*NFKBIA*, *NFKBIB*, *NFKBIE*, *NFKBIZ*, *BCL3)* were rapidly induced by LPS, as were genes for molecules such as *TNFAIP3* (A20) which mediates feedback inhibition of NFκB through regulated ubiquitination. *OTUD1* encodes a novel inducible deubiquitinase (DUB) enzyme, in the same structural class as *TNFAIP3* but not previously implicated in control of the LPS response. Other induced genes for feedback repressors of the initial signaling cascade include the protein tyrosine kinase *LYN*, multiple members of the TRIM family of E3 ubiquitin ligases which promote degradation of signaling molecules (*TRIM5*, *TRIM10*, *TRIM25*, *TRIM35*, *TRIM36*, *TRIM38*), the TRAF inhibitor *TANK*, multiple inhibitor microRNAs (notably *miR146A*, *miR155*, *miR21*, *miR3648*, *miR4741*), *IER3*, each of the GADD45 family members, which amongst many other targets, probably inhibit p38 MAP kinases [64], the caspase inhibitor *TNFAIP8* and the transcription factor *ATF3* [28]. PPP1R15A (also known as GADD34), a regulator of protein phosphatase 1 is identified as a feedback regulator of TLR-induced phosphorylation of TAK1 [65]. One gene of unknown function that was clearly induced was *ZBTB10* (*RINZF*), most likely also encoding a transcriptional repressor [66].

Many proinflammatory genes encode short-lived mRNAs with AU-rich elements in their 3’UTR, subject to degradation by the *ZFP36* (tristetraprolin) gene product [62,67]. Two related genes, *ZFP36L1* and *ZFP36L2*, have also been implicated in control of mRNA stability in LPS-stimulated mouse macrophages [68]. However, by contrast to mouse macrophages, in human MDM, ZFP36 is repressed by CSF1 (compared to high levels in monocytes), and none of the ZFP36 family genes was highly-inducible by LPS. Instead, *ZC3H12A*, another gene encoding a novel ribonuclease that controls stability of other inflammatory cytokine mRNAs, notably *IL6* and *IL12p40* [69], was highly-induced by LPS, within a similar time course to *TNF*, but remained elevated even after 48 hours. Interestingly, *PARP14*, encoding a member of a family of intracellular proteins that generate ADP-ribose posttranslational adducts, was strongly induced by LPS, commencing around 2 hours, and peaking at 12 hours (within Cluster 3). PARP14 forms a complex with ZFP36 and the AU-rich element in the mRNA 3' untranslated region of the tissue factor (*TF*) gene [70]. The neighbouring *PARP15* and *PARP9* genes were also induced by LPS. *PARP9* shares a shares bidirectional promoter with the E3 ubiquitin ligase gene *DTX3L*, and the two proteins interact to control, amongst other things, the function and expression of *IRF1* [71]. Other members of the PARP family, *PARP7*, *PARP10* and *PARP12*, which control protein translation [72] and/or feedback inhibit NFκB signaling [73] were also LPS-inducible at later time points. Based upon the function in mice, the inducible expression of the signaling molecule gene, *IRAK2* [74] and the regulator of intracellular trafficking, optineurin [75] probably contributes positively to the sustained induction of inflammatory genes.

*HCAR2* and *HCAR3* (also known as *GPR109A* and *GPR109B*) are neighbouring duplicated genes in the human genome, and encode receptors for butyrate and niacin. Both genes were induced in parallel by LPS. In mice, GPR109A has been associated with feedback regulation of the LPS response and suppression of macrophage reactivity to gut luminal contents [76]. *PELI1* (Pellino1) encodes an E3 ubiquitin ligase that is required for TRIF-dependent signaling from TLR3 and TLR4 [77] and probably enables subsequent induction of the IFN target genes. *XBP1* lies downstream of the gene for the ER stress sensor kinase, IRE1alpha (*ERN1*), and in mice XBP1 was found to be required for optimal and sustained cytokine production by macrophages responding to LPS [78]. The gene for another target of ER stress, *PPP1R15B* [79] was induced in parallel with *XBP1*. *MSC*, encoding musculin or activated B cell factor-1 (ABF-1) [80], a repressor of bHLH transcription factors in muscle and B cells which has not previously been reported in macrophages, was also within this cluster.

One novel finding was the marked induction of *PDSS2*. *PDSS2* encodes prenyl (decaprenyl) diphosphate synthase, subunit 1, an enzyme involved in the synthesis of coenzymeQ (CoQ). It is not known whether the activity of this enzyme limits flux through the pathway, but heterozygous mutation in another gene in the CoQ pathway, *Mclk1* (*Cog7*) in mice, produced increased levels of TNF in macrophages, and hypersensitivity to LPS [81]. Hence, this gene probably also contributes to feedback inhibition of the LPS response.

The autocrine interferon response induces its own set of feedback regulators, notably the obvious suppressors of cytokine signaling (*SOCS1*, *SOCS3* and *SOCS6*) which were each induced with distinct time courses (S2 Table). *PLEKHF2* is linked to control of interferon production [82]. LPS-inducible *GPR183* (also known as *EBI2*, or EB virus induced 2) is a feedback regulator of type 1 interferon pathways [83]. The ligand for EBI2 is 7α,25-dihydrocholesterol and the enzyme that synthesises it, *CYP7B1*, was also induced strongly by LPS.

Finally, the induction of the metal ion responsive transcription factor, MT1, is linked to the recently described role of zinc in feedback regulation of NFκB activation and inflammatory transcription [84]. Indeed, genes for the zinc transporters, *SLC39A8* (ZIP8), *SLC39A14* (ZIP14) and *SLC30A1* (ZNC1), were each strongly induced by LPS, commencing from around 4–5 hours after induction, and rising continuously.

### The activation of enhancer-associated transcription in response to LPS precedes target gene activation

LPS stimulation of mouse macrophages was reported to induce transcription from some 3000 enhancer loci, preceding modification of histone methylation [31,85]. eRNAs are relatively unstable, and are degraded by the RNA-exosome complex [86]. However, CAGE enables their quantitative detection, and the activation of eRNA transcription can be correlated with subsequent activation of promoter activity in putative target genes in the chromosomal vicinity [35]. Many enhancers described by the FANTOM5 consortium came from stimulated monocytes. The genes encoding limiting exosome components, *EXOSC3* and *EXOSC10* were low in MDM, and repressed transiently between 1 and 2 hours after LPS stimulation. CAGE tags derived from the genes encoding several other exosome components, *EXOSC1*, *EXOSC2*, *EXOSC5* and *EXOSC7*, were almost undetectable in monocytes in culture. The relative lack of the exosome complex may facilitate the detection of active enhancers in monocytes and macrophages.

S2 Fig shows profiles of enhancer activation at a selected subset of inducible genes in which there was robust activation of transcription that clearly preceded the appearance of transcripts from associated promoters. In every case, the enhancers that have been identified in the time series are robustly supported by evidence of inducible bi-directional transcription in the LPS-stimulated monocytes. The data strongly support the view that enhancer transcription precedes activation of target promoters [35].

**Fig 3:**
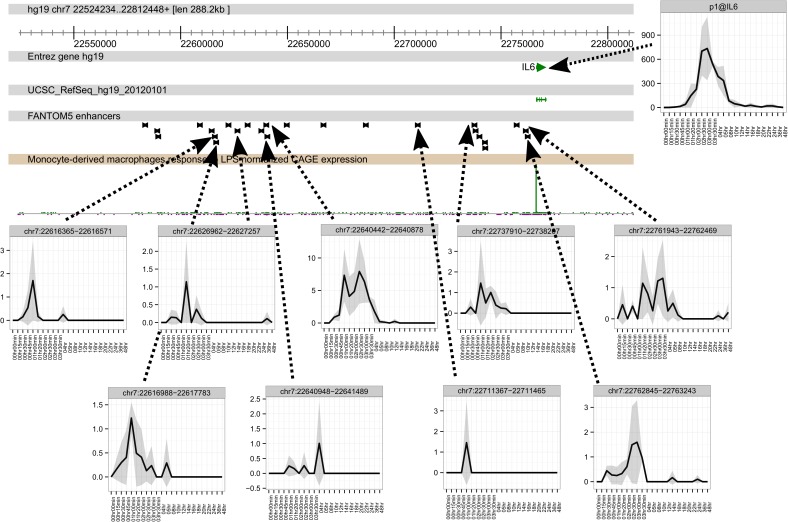
The time course of activation of enhancers and promoter at the IL6 locus. The core panel shows a genome browser view of the *IL6* locus with the locations of FANTOM5 enhancers. The upper right panel shows the time course of induction of *IL6* mRNA, detected by CAGE, which peaks around 3–4 hours and declines by 12 hours. The lower panels show the transient activity of the enhancers indicated, the majority of which peak around 1–2 hours and decline rapidly. Data are expressed at TPM, and are the average of the three replicates.

In many of these cases there were numerous individual regulated enhancers apparently associated with a single inducible promoter. For example, the *IL6* locus has recently been dissected in detail in human monocyte-derived macrophages responding to LPS [56], showing the roles of STAT1 and IRF1 in establishing permissive chromatin architecture in the vicinity of the gene, and identifying sites up to 50kb upstream of the transcription start site. The FANTOM5 data for the LPS induction series revealed LPS-inducible bidirectional transcription up to 150 kb upstream of *IL6*. Fig 3 shows a time course of detection of transcription of each of these elements; nine separate elements had detectable activity in LPS-stimulated MDM in advance of the peak of *IL6* promoter activity. These elements were more readily detectable in LPS-stimulated monocytes, where almost all of the 25 detected enhancers had significant transcriptional activity.

**Fig 4:**
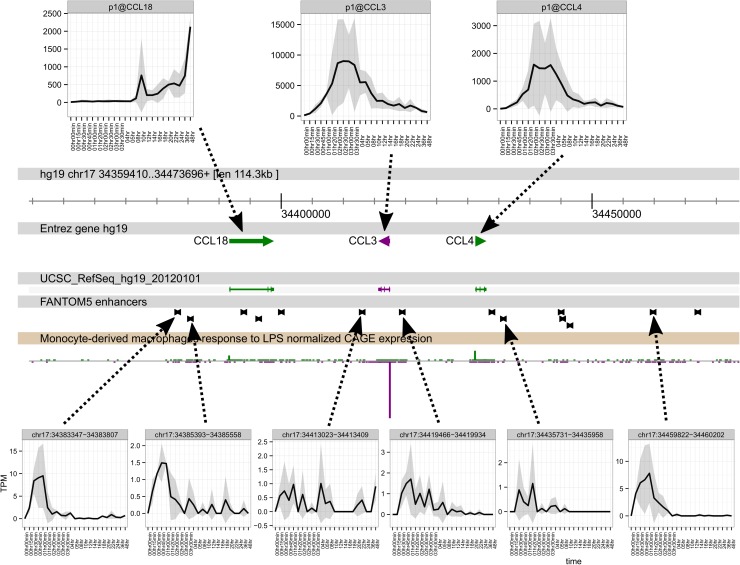
The time course of activation of enhancers and promoters at the *CCL3/CLL4/CCL18* locus. The core panel shows a genome browser view of the locus with the locations of FANTOM5 enhancers. The upper panel show the time course of induction of each of the mRNAs, detected by CAGE. Whereas *CCL3* and *CCL4* are coordinately-regulated, *CCL18* follows a much slower time course and is still rising at 48 hours. The lower panels show the activity of the enhancers indicated. The lowest track shows the histograms of CAGE tags mapped to the region, with colours indicating direction of transcription; green to the right and purple to the left. Note that the entire regions shows evidence of bidirectional transcription initiation. Data are expressed as TPM, and are the average of the three replicates.

**Fig 5:**
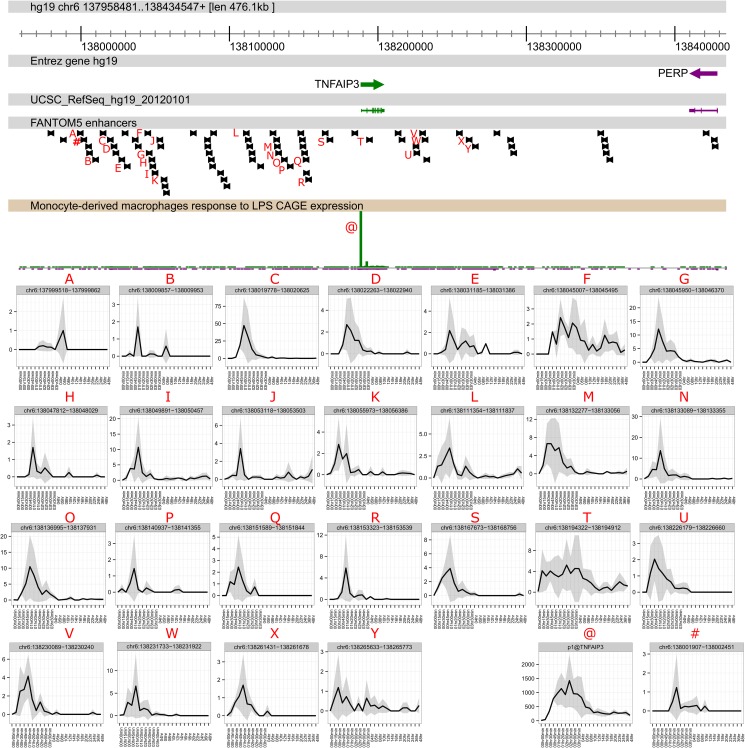
The time course of activation of enhancers and promoter at the *TNFAIP3* locus. The core panel shows a genome browser view of the TNFAIP3 locus with the locations of FANTOM5 enhancers. The lower right panel (p1@TNFAIP3) shows the time course of induction of *TNFAIP3* mRNA, detected by CAGE, which peaks around 2 hours and declines to a new, elevated steady state by 8 hours. Other panels show the transient activity of the enhancers indicated, the majority of which peak around 1–2 hours and decline rapidly. Panel at bottom right shows the activity of the enhancer containing the SNP originally associated with CD susceptibility, 185kb upstream of the *TNFAIP3* locus [2]. Data are expressed as TPM, and are the average of the three replicates.

The chemokine genes *CCL3* and *CCL4* are coregulated by LPS. Here again, there was bidirectional promoter activity associated with enhancers outside, and between, the two inducible genes, more readily detected in the LPS-stimulated human monocytes (Fig 4). Two enhancers downstream of *CCL4* (to the right of the panel) were induced ahead of coordinated induction of *CCL3* and *CCL4* promoter activity. By contrast, the neighbouring *CCL18* gene was much more slowly induced, and four enhancers appeared to show more prolonged activation. The entire region shows evidence of bidirectional promoter activity in LPS-stimulated monocytes. Finally, the region surrounding the *TNFAIP3* locus contained an array of enhancers extending over 400kb, at least 25 of which had detectable induction of transcription in advance of the activation of the promoter (Fig 5).

A recent study identified around 120 long non-coding RNA (lncRNA) that were induced by LPS in human monocytes based upon RNAseq [87]. We reexamined these loci individually in the FANTOM5 data. All but one of the top 20 most-inducible candidate lncRNA were contained within large clusters of enhancers (so-called super-enhancers) identified by the FANTOM5 data, and were actually associated with bidirectional transcription in LPS-stimulated monocytes. The potential target loci identified included *IL6* and *TNFAIP3* (discussed above), as well as *IDO1*, *miR155*, *ACSL1*, *IRF2*, *HS3ST3B1*, *TNFSF8*, *DDX58*, *CD38* and *SLAMF7*. The only exception is *NONCO3094*, which is driven by a strongly-LPS-induced promoter that is antisense to *IL7* (which is not itself expressed in monocytes/macrophages). Based upon the extensive linked CTSS associated with enhancers in these regions, there is some question as to whether the proposed lncRNA could actually be artificially concatenated over-lapping short RNAs.

### Global analysis of the transcriptional cascade from motif activity response analysis

**Fig 6:**
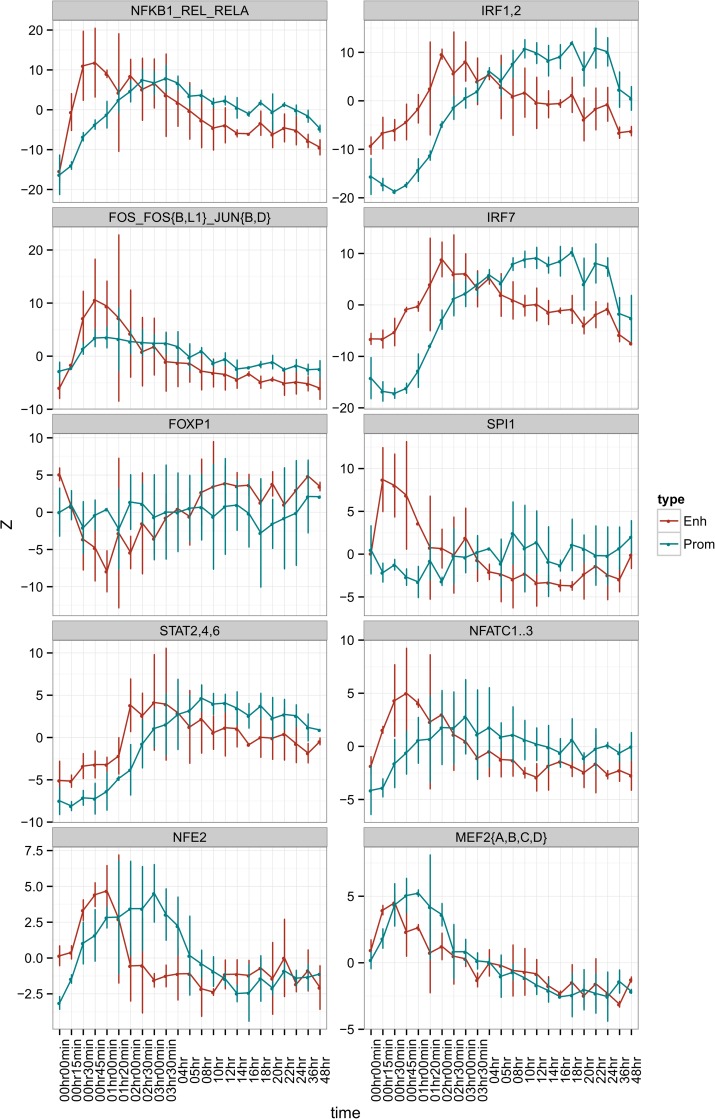
Motif activity response analysis (MARA) of the response to LPS MARA analysis of promoters and enhancers was carried out as described in Materials and Methods. MARA averages the expression of all promoters (blue) and enhancers (red) that contain the consensus transcription factor binding motif. Activities are plotted as Z-scores (averages of activity divided by standard deviation) in each time point, with error bars representing the standard deviation of Z-scores.

When sets of co-regulated genes share a particular motif in their promoters, we can infer that DNA binding protein(s) that recognize the motif regulate that set of genes. We utilized Motif Activity Response Analysis (MARA) [88] to identify transcription factor binding motifs associated with the response of the MDM to LPS. Fig 6 shows the motif activities for the most active motifs. There was a temporal separation of motif activities associated with enhancers (red lines in Fig 6), which peaked before the activation of promoters through the same motifs (blue lines in Fig 6). The exception to this pattern was the macrophage-specific transcription factor, *SPI1* (PU.1), which showed early motif activity for enhancers, but no subsequent activation on promoters. This finding is consistent with the proposed role for PU.1 as a pioneer transcription factor that defines “latent enhancers” that are subsequently bound by other factors in response to agents such as LPS [32,89].

The earliest response detected by MARA was associated with the Fos/Jun, or AP1 motif, consistent also with these factors being amongst the earliest targets of LPS activation (see above). Subsequently, the increased activity of the NFKB/REL/RELA motif peaked around 2 hours, but declined only slowly (Fig 6). An initial burst of activity probably derives from the translocation of a preexisting complex subsequent to activation by IKB kinases [27]. Subsequently, from around 2 hours, genes encoding all of the members of the NFκB family, *REL* (c-rel), *RELA* (p65), *RELB*, *NFKB1* and *NFKB2*, were induced and presumably contributed to both replacement of the cytoplasmic pool of NFκB and transcriptional regulation of a distinct late-response set of target promoters containing the NFKB/REL/RELA motif. The progressive increase in motif activity of IRF1/2, IRF7 and STAT2,4,6 was initiated later in the time course, and is entirely consistent with the induction of IFNB1, and of the IRFs themselves, as discussed above.

The MARA analysis reinforces the likely importance of the stress response in LPS action. NFE2 (also known as NRF) sites were previously identified as active motifs in LPS-stimulated mouse macrophages [30]. The stress response factors, *NFE2L1* (*NRF1*) and *NFE2L2 (NRF2)* are the likely occupiers of the NRF sites, being expressed constitutively in MDM but further inducible by LPS. The motif annotated as NFATC (1…3) is most likely bound by *NFATC1*, which was induced by LPS, peaking at 2 hours. *NFATC1* has two promoters encoding alternative 5’ exons in both mouse and human, as discussed in a recent review [90]. The more distal promoter was stimulated by LPS. *NFATC1* is itself a downstream target of both AP1 and NFκB factors [90].

MARA also implies a function for the MEF2 family. *MEF2A*, *MEF2C* and *MEF2D*, which recognise the MEF2 motif, were all expressed constitutively in MDM, as described by others [91] and the latter two were down-regulated by LPS late in the time course. Like NFκB these proteins undergo regulated nuclear translocation [92]. MEF2 most likely underlies the motif activity attributed to the serum response factor SRF (another MADS box protein). Although there have been reports of the activity of SRF in mouse macrophages [93] *SRF* was expressed at barely-detectable levels in MDM, similar to the Ets components of the serum-response element complex (*ELK1*, *ELK3*, *ELK4*).

The absence of detectable motif activity based upon MARA does not constitute evidence that any particular factor does not contribute to transcriptional regulation. The GGAA/T core motif bound by ETS family factors is relatively uninformative, and overlaps with the purine-rich PU.1 motif found in many myeloid-specific promoters [16]. Genes encoding ETS factors with related binding motifs, including *ETS2*, *ELF4*, *FLI1*, *ETV3*, *ETV5* and *ETV7* (TEL) were each up-regulated by LPS with distinct kinetics. Sweet et al. reported previously that *ETS2* is not only induced, but is phosphorylated on the pointed domain, required for effective trans-activation, in response to LPS [94].

MARA is also rather insensitive for the detection of activity of factors that bind GC-rich motifs within CpG island promoters. For example, the “KLF4” motif (CaCaCCC) showed little change in activity across the LPS time course. However, as evident from the clusters in Fig 1, multiple members of the KLF family were regulated in response to LPS. *KLF2* was one of the immediate early genes. *KLF7* has two promoters, one induced by 2 hours, another induced later in the time course. *KLF5* was induced even later (around 3–4 hours, cluster 43). *KLF3* also has two promoters, one induced at 7–8 hours, one at 20 hours. Each of these KLF family members could have a distinct function and distinct target genes. In mice, *KLF4* was suggested to be a feedback regulator, and to polarise macrophages towards M2 phenotype [95]. In humans, *KLF4* was constitutively expressed in monocytes and almost completely repressed in MDM. It was transiently, but relatively weakly-induced by LPS. *KLF6* did not cluster in the coexpression analysis, having an idiosyncratic expression pattern. It was induced rapidly, peaking by around 80 mins in all samples, but unlike most early response genes, it declined slowly and remained somewhat elevated even after 48 hours. *KLF6* is in a rather gene poor-region of the genome, and is surrounded by numerous enhancers identified in the FANTOM5 dataset. Of these, at least 4 were detectably and transiently-induced ahead of the peak of accumulation of *KLF6*. KLF6 is required for optimal LPS-induced gene expression in mice [96].

### Macrophage-specific gene expression and inflammatory bowel disease susceptibility

**Fig 7:**
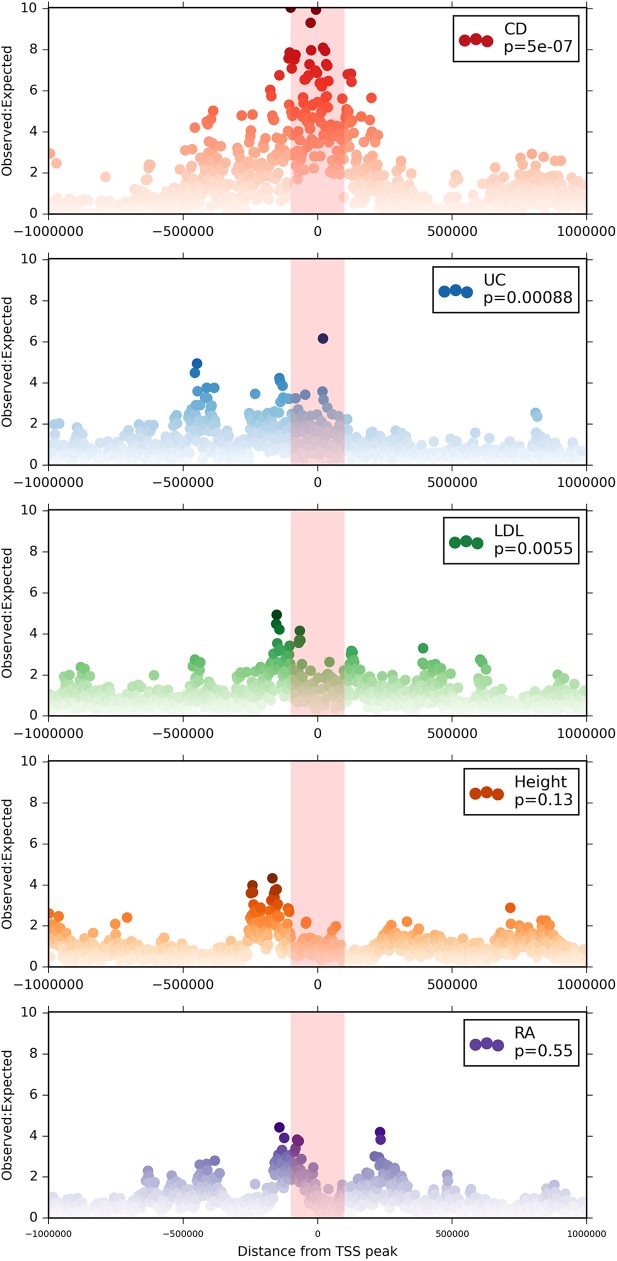
Enrichment of macrophage-expressed or regulated genes in genomic regions associated with IBD susceptibility Each panel shows the enrichment of GWAS-associated variants in vicinity of genes meeting the expression criteria (specifically inducible in monocytes AND down regulated during differentiation to macrophages) for each of the traits/diseases shown. For 1000 bins spanning a region of 1Mb above and below all TSS meeting expression criteria, observed:expected ratios were calculated as the ratio of the absolute count of variants with p<1e06 for association with each trait, to the absolute count of all SNPs genotyped in the same study. p-values for enrichment were calculated for the whole set using PASCAL (see Methods).

Based on the observations described above, we hypothesised that IBD-associated genes, of which NOD2 is an archetype, are likely to be specifically-inducible in monocytes in response to inflammatory stimuli AND down-regulated during differentiation to macrophages. In order to test this hypothesis, we quantified the evidence for disease association in the regions surrounding promoters meeting these criteria. 2413 promoters exhibited this expression pattern, out of a total of 201,801 promoters in the FANTOM5 dataset. After correcting for linkage disequilibrium, regional co-regulation, and the genomic distribution of variants (see Methods), we found that variants associated with Crohn’s disease by GWA were very strongly enriched in the regions surrounding this set of promoters (Fig 7). Less enrichment was seen for genes putatively associated with ulcerative colitis, and no significant enrichment was observed for control sets of loci/SNPs associated by GWA with the other traits (rheumatoid arthritis (RA), LDL cholesterol, and height). The association between CD loci and regulated transcription in macrophages can also be visualized by examining the expression profiles of promoters that lie within 2kb of a putative Crohn’s disease-associated SNP (p<10e-6). This subset is displayed in Fig 2B.

To enable visualization of the results of the CAGE data with the location of disease-associated SNPs, we created a genome viewer that displays locations of FANTOM5 promoters and enhancers together with the P values for association with CD and UC and other traits (http://gsht.baillielab.net; available for review; this resource is currently being transferred to a permanent server, which will be available at the time of publication. We also provide a direct link to visualize the association data on the ZENBU browser (see Methods).

The most extensive meta-analysis of IBD GWA data to date [4] identified 163 loci that met genome-wide significance thresholds for CD, UC or IBD. A subsequent study identified an additional 38 loci that underlie shared genetic risk across different ethnicities [5]. Most dissections of candidate genes in GWAS focus on the nearest gene to the peak of association, or the genes within the genomic interval of association, and assume that actual causal variants are in high LD with the SNP(s). The genomic intervals are not always precise, and of course they may contain enhancers that alter transcription of genes outside that interval.

The criteria we tested (specifically inducible in monocytes; down-regulated during differentiation to macrophages) identify plausible alternative candidates in many known IBD loci. For example, the *MUC19* gene on Chr12 is annotated by Jostins et al. [4] as the only gene with the IBD GWA candidate interval defined by SNP rs11564258. However, Lacour et al. [97] identified an extended haplotype that includes the neighbouring *LRRK2* gene, a clear candidate for which there is already functional evidence of a role in IBD [98]. *LRRK2* is identified by our criteria, and shares transcriptional regulation with *NOD2*, being expression and further induced by IFNγ in monocytes and ablated by CSF1 in monocyte-derived macrophages.

S3 Table provides a detailed manual curation of genes within >200 genomic intervals from published IBD datasets, inspected using the viewer described above, and gene expression derived from the FANTOM5 data. All but 12 of the intervals within the boundaries of significant GWA peaks for IBD contained promoters/genes for which expression was strongly monocyte-macrophage-enriched and/or regulated by CSF1, LPS or both. For some regions identified by GWA, no candidate gene had been proposed but the CAGE data implicate a novel candidate that was expressed and/or regulated specifically in monocytes or macrophages or both. For others, our analysis identified alternative candidates to those that are currently emphasized. For example, *NOD2* was regulated in parallel with two flanking genes, *SNX20* and *CYLD* (cylindromatosis). These three genes are close to monocyte-specific, IFNγ -inducible enhancers, detected by CAGE, located in the interval between *NOD2* and *SNX20*. All three genes were expressed highly in monocytes and almost absent from MDM grown in CSF1. SNX20 has been implicated in control of P-selectin location, and might therefore also contribute to extravasation of monocytes [99]. *CYLD* encodes a deubiquitinating enzyme that exerts feedback control on both NFκB and MAP kinase pathways and has itself been implicated in inflammation control [100]. So, even in this archetypal CD susceptibility locus, NOD2, the gene that has been most studied, is not necessarily the best or only candidate, notwithstanding evidence of protein-coding variation.

Another well-studied candidate IBD susceptibility gene, *ATG16L1*, was expressed ubiquitously in the FANTOM5 atlas, providing no support for a gut-specific or inflammatory pathology. By contrast, the neighbouring gene, *INPPP5D* (inositol polyphosphate 5-phosphatase, also known as *SHIP1*), shares tight co-regulation with *NOD2*, being high in monocytes, induced by IFNγ, down-regulated in MDM, and further ablated by LPS. Mice deficient in SHIP1 have chronic inflammatory disease, including a Crohn’s-like colitis [101]. A recent detailed analysis of the *ATG16L1* association with IBD revealed that the commonly-studied SNP is in linkage disequibilibrium with SNPs across an extended haplotype and additional variants could implicate any region of the locus [102]. The CAGE data also identified at least four monocyte-specific, regulated enhancers in the 40kb interval between *ATG16L1* and *INPPP5D*, including one only 2.5 kb upstream of the *ATG16L1* locus (Chr2:234156397). Immediately downstream of *ATG16L1*, another candidate within the genomic interval identified by linage to CD is *DGKD*, diacylglycerol kinase delta, also expressed highly in monocytes and ablated completely in MDM. The hypothetical involvement of dysregulated autophagy in IBD [4], is based largely on association with *ATG16L1* and is cast into doubt if this gene is not the only, or even the most likely, candidate gene in the region.

The association of IBD susceptibility with autophagy also rests in part upon genetic linkage to *IRGM*, which has been linked in turn to the functions of the large family of inducible GTPases in the mouse. The original study of human *IRGM* [103] refers to the gene as an ortholog of mouse *Irgm1*, but the location of human *IRGM* in the genome is actually not syntenic with mouse (http://www.ensembl.org). Humans have lost the inducible GTPase family as a mechanism of host defense, and *IRGM* was shown not to be interferon-inducible in humans [104]. The evidence that the human *IRGM* locus actually encodes an expressed transcript or a functional protein that is detected in any cell type is equivocal [105]. Strongly indicating that it does not, the FANTOM5 CAGE data detects no expression of *IRGM* mRNA in any cell population or tissue in any state of activation. The *IRGM* SNP associated with IBD susceptibility is in strict LD with a 20kb deletion polymorphism, 2.5kb upstream of the putative TSS of *IRGM* [106]. One alternative explanation for the association is that the *IRGM* region contains distal enhancers for macrophage-expressed genes. Chromatin immunoprecipitation sequencing (ChIPseq) data derived from monocytes and macrophages suggests this is the case, with clear peaks for binding of PU.1 and enhancer marks [20] corresponding to a cluster of enhancers identified by CAGE, which can be visualised on the ZENBU browser. Amongst the neighbouring genes, *SMIM3*, encodes a small integral membrane protein about which little is known, but it is up-regulated in MDM and further induced by LPS. The most likely candidate is *TNIP1*, encoding a regulator of NFKB signalling, which has been implicated in genetic susceptibility to other inflammatory diseases [107].

The association of the *NKX2*.*3* locus on chromosome 10 with IBD susceptibility has been widely-reported and replicated, but no function of the gene product associated with inflammation has been described. Within the same region, *SLC25A28* encodes a mitochondrial iron transporter (also known as mitoferrin 2) which showed the same pattern of regulation as *NOD2*, down-regulated in MDM compared to monocytes, but very strongly-induced by LPS. Similarly, no mechanistic link has been made between the SNP associated with the *IL23R* locus and IBD susceptibility, but there has been considerable emphasis in the literature on the central role of Th17 T cells, activated by IL23, in disease pathogenesis [4]. There is an alternative myeloid-expressed candidate gene, *MIER*, within the linked candidate interval surrounding *IL23R*.

Previous data have revealed that promoters and enhancers identified by CAGE are strongly enriched for informative SNPs in GWAS [35]. One specific example is the *IL12B* locus, where the SNP rs6871626, associated with CD susceptibility, is located within one of a cluster of LPS-inducible upstream enhancers. Interestingly, susceptibility to tuberculosis in an African cohort has been associated with a separate SNP cluster 3’ of the *IL12B* locus and of the neighbouring *UBLCP1* gene ([108]). The *UBLCP1* gene is expressed ubiquitously, but these remote enhancers were activated by LPS in both monocytes and MDM. Similarly, Jiang et al [109] identified multiple allelic variants in the *PTGER4* locus on chromosome 5, that associated independently with CD. The FANTOM5 data revealed around 50 LPS-inducible enhancers in the 0.5Mb upstream of *PTGER4*. Confirming this pattern, the subset of CTSS within 2kb of a putative CD-associated promoter has a distinct pattern in the LPS time course data.

Prior to the adoption of population GWAS, family-based studies identified six IBD loci with very high relative risks amongst related individuals. IBD1 contained *NOD2*, and IBD3, the well-known association with the complex HLA locus. IBD5, located around chromosome 5q31, was located to a missense mutation in the organic cation transporter, *SLC22A4* [110] and a promoter variant in the neighbouring *SLC22A5* gene. The combined haplotype conferred a 7.5 fold risk of Crohn’s disease [111]. Consistent with our hypothesis, *SLC22A4* was highly-expressed in CD14^+^ monocytes, and completely down-regulated by CSF1. The IBD6 locus was located on Chromosome 19p13, by analysis of patients/families that did not possess *NOD2* variants [112]. Subsequent analysis revealed multiple variants associated with the linked *EMR1*, *EMR2*, *EMR3* and *CD97* cluster of genes that contributed to the overall IBD susceptibility. All four of these genes were expressed at high levels in monocytes, and strongly down-regulated by CSF1. The strong expression of *EMR1* contradicts previous report claimed that human *EMR1* is expressed only in eosinophils [113]. *Emr1* in mice encodes the F4/80 antigen, widely used as a macrophage marker [113]. The expression of *EMR1* in humans is clearly different in mice, where the mRNA and protein are actually induced by CSF1 and are retained on tissue macrophages. However the phenotype of a knockout of the gene in mice implies a function in oral and peripheral tolerance [114].

To further explore candidate loci, we considered one individual component of the criteria described above that is central to our hypothesis: the large set of promoters that is expressed in freshly-isolated blood monocytes (excluding those that were isolated by adherence and culture *in vitro*) and either up or down-regulated at least 5-fold in monocyte-derived macrophages grown in CSF1. These promoters and their expression values in all samples analysed are shown in S4 Table; in many cases we identified multiple promoters from the same locus, as well as distal enhancers that have not been ascribed the gene name, but may be connected based upon their apparent strict coexpression [35]. Excluding existing candidate genes such as NOD2/SNX20, amongst the promoters down-regulated by CSF1 in MDM, and associated with specific gene names, at least 88 genes were identified as functional candidates lying within 200kb intervals with a peak p value of at least 10^−6^. These are annotated in S4 Table. Amongst these new candidates, several transcription factors (*JUN*, *FOS*, *FOSL2*, *JUND*, *ETS1*, *ETS2*, *NFKB1*, *NFKBIZ*, *NFKBIA*, *CREM*, *SMAD3*, *BATF3*, *NFAT5*, *NR1D1*, *RARA*, *NFACTC4*, *FOXP1*, *PRDM1*, *NFIL3*, *KLF3*) are of particular interest since they are likely to produce *trans-*acting impacts. Note that CSF1 strongly down-regulates many of the non-MHC genes within the HLA region on chromosome 6 (and to a lesser extent, most Class II MHC (HLA-D) transcripts) providing an alternative explanation for some of the well-documented HLA association with CD. Amongst the CSF1-inducible genes annotated in S4 Table there were a further 46 candidates. This set did not include any transcription factors, and in the large majority of cases, the association was specific to either CD or UC. In passing, we noted that the CSF1-inducible set separately identifies many genes, including CSF1 itself, with loci associated with lipid traits (e.g. LDL cholesterol), and as previously noted [115], CSF1 induced many genes associated with lipid metabolism. The IBD loci identified by Jostins et al. [4] do not include any on the X chromosome, which is consistently under-represented in GWAS [116]. Nevertheless, there is evidence for X-linked IBD susceptibility [117]. Amongst the genes on the X chromosome, *TLR8* is strongly monocyte-specific, and is part of an extended haplotype associated with IBD [118]. *ARHGEF6*, also linked to CD [116], is also strongly monocyte-enriched and expression was abolished in MDM.

In summary, on the sole basis of regulation in monocytes by CSF1 as a biological prior, we identify a further 134 candidate genes associated with IBD susceptibility, in addition to novel candidates within IBD loci identified from traditional linkage analysis. S4 Table contains many additional regions that are not associated with a gene name, and the larger set of 2400 promoters analysed in Fig 7 (S5 Table) contains 392 named genes that will undoubtedly expand the list of candidate functional loci still further.

## Discussion

### The monocyte-derived macrophage response to LPS as a model for intestinal macrophage differentiation

We analysed the response of human macrophages, differentiated in CSF1, to the TLR4 agonist LPS in order to determine whether this mirrors the development of gut macrophages and hence whether gut macrophages might be involved in the development of IBD. The direct relevance of this system to IBD is reinforced by the recent identification of a functional missense variant in the LPS receptor, TLR4, associated with Crohn’s disease [2].

We have produced an unprecedented analysis of the transcriptional events during the response of macrophages to LPS. Figs 1 and 2 and S1 Fig overview the complex transcriptional cascade of feed-forward and feedback regulation of sets of genes so that the transcriptome is not stable even after 48 hours. The earliest events detected were the induced transcription of enhancers ([37] (S2 Fig). As a class these regions are opened by binding of pioneer transcription factors. In the case of macrophages, the dominant pioneer is PU.1 [20,89,119,120]. The observation that enhancer transcription precedes activation of target promoters is known from other systems, and indeed one might use this correlation to infer the likely connection between distal enhancers and promoters [35]. What is less obvious in all of the examples shown in Figs 3, 4, 5 and S2, is that the induction of enhancer activity appears transient, even when the putative target gene is induced relatively stably. The transcripts are presumably rapidly degraded by the exosome complex. This suggests that the act of transcription of an enhancer is associated with its activation, but the transcript *per se* is less likely to be functional.

Subsequent to early enhancer activation, wave after wave of transcription factors was induced (Fig 1) followed by their putative target genes (Fig 2) which were identified by enrichment of target motifs in their promoters (Fig 6). Genes for the inducible transcription factors, such as the members of the IRF family, formed clusters with their likely target genes, which contained binding site motifs detected by MARA. The temporal data provide indications of distinct and specific roles. For example, distinct members of IRF family were found in different clusters whereas in a previous analysis of monocyte eQTL data, *IRF7* and *IRF9* clustered together [47].

The endpoint of differentiation of mucosa-associated macrophages is a population that is longer responsive to restimulation with LPS or other microbial challenges [121]. The FANTOM5 data demonstrate that MDM grown in CSF1, in common with mucosal macrophages, strongly down-regulated most pathogen recognition molecules expressed at high levels in monocytes, notably *TLR1*, *2*, *4* and *6*, *NOD2* and other Nod-like receptors (NLR) and non-NLR inflammasome activators (*NLRC3*, *NLRC5*, *NLRP1*, *NLRP3*, *NRLP6*, *MEFV*, *PYCARD*), C-type lectins *CLEC7A* (dectin1), *CLEC5A*, *CLEC4D*, *CLEC4E and CLEC12B; SIGLECs 3*, *5*, *9*, *10* and *14*, formyl peptide receptors *FPR1* and *FPR2*, recently described cytoplasmic LPS receptors *CASP4* and *CASP5* [36] and the nucleic acid detector *CGAS*. There is clearly the potential for dysregulation of any of these individual pattern recognition receptors, which could explain why it has been difficult to link IBD incidence to particular microbial challenges common to all patients [1,122].

Compared to monocytes, MDM also shared with human gut macrophages the suppression of *CD14* (the LPS coreceptor), and adaptor and signaling molecules such as *MYD88*, *TRIF*, *TRAF6* and *IRAK1*. So, although they clearly do respond to LPS, the response of MDM is greatly attenuated compared to the response of blood monocytes. The FANTOM5 data assays the blood monocyte response to LPS at only a single time point, but based upon the quantitative measure of CAGE tag frequency, the classical inflammatory cytokines, *IL1B* and *IL6* were each induced by LPS to levels 10–100 fold greater in monocytes than at any time point in the MDM time course. By contrast, the monocyte attractants, *CCL2*, *CCL3* and *CCL4* were induced to comparable levels in MDM and monocytes. We suggest that once inflammation has been initiated through the activation of hyper-responsive incoming monocytes, or resident macrophages, accelerated recruitment of further monocytes would induce a self-perpetuating hyperinflammatory state. TGFB1 has also been implicated in mucosal macrophage differentiation, and strongly implicated in IBD [123]. The modulated response of MDM to LPS probably involves this factor. The MDM are most likely autocrine for TGFB1 signaling, since they express high levels of *TGFB1* mRNA, and both receptors, *TGFBR1* and *TGFBR2*.

### Extensive feedback control of the LPS response

The rigorous feedback control that is documented here has previously been seen as a way to produce robustness and stringent control [24]. If this were true, one might expect the system to be resilient in the face of varied expression or function of individual components. This is clearly not the case. Most of the literature on the function of inducible repressors derives from studies of inbred mice, in which knockout of any one these regulators produces dysregulated LPS responses [24,25,83]. Parnas et al. [124] reported a genome-wide CRISPR screen that revealed hundreds of genes apparently required for the production of TNF by mouse macrophages in response to LPS. The structure of the screen did not reveal as many genes involved in negative regulation. To gain further insight into the scale of feedback control of the response, we searched PubMed to identify mouse knockouts that impact the response to LPS. S6 Table identifies >180 additional mouse loci where a null mutation is viable but produces a global change in macrophage responsiveness to LPS *in vitro* and/or *in vivo*. The list does not include all of the inflammatory cytokines and chemokines, and essential signaling molecules. Almost 2/3 of these mutations produced hypersensitivity to LPS administration or sepsis *in vivo*, and many also produced spontaneous colitis. The set of LPS susceptibility genes in S6 Table is annotated with the expression from the FANTOM5 data, and excludes the obvious signaling molecules and inflammatory cytokines and chemokines in the KEGG Toll-like receptor signaling pathway (hsa04620). In every case, the FANTOM5 data confirms that the genes are also expressed/enriched in human monocytes and/or regulated by CSF1, LPS, or both. All of this discussion indicates that there may be hundreds of different genomic variants that can each contribute to an increased susceptibility to IBD.

### The functional genomics of IBD susceptibility

A major limitation of GWAS is the assumption that common variants underlie the risk of disease in different families. The hypothesis-free GWA approach can drive understanding of new areas of biology. Conversely, we have shown clearly in Fig 7 that a biological prior, based upon transcriptional co-expression, in this case based upon expression and regulation in monocytes, can focus analysis on a subset of genomic regions and specific candidate genes within those regions, thereby reducing the false discovery rate and the genome-wide significance threshold. Our hypothesis reduced the number of candidate genes/promoters analysed by two orders of magnitude (around 2000 of 200,000 promoters). A previous study aimed at identifying gene features linked to CD susceptibility noted the differential expression of genes associated with “M1” versus “M2” macrophage activation states as a criterion [12]. The definition of those activation states is somewhat problematic [125,126]. We have taken a more nuanced view, with a much more extensive dataset, to emphasise the specific importance of CSF1 and monocyte differentiation.

*NOD2* is amongst the most studied of loci underlying Crohn’s disease susceptibility, and there are several protein-coding variants apparently linked to disease. However, there is little consensus about NOD2 function in the GI tract and the mechanistic link between sequence variants and disease susceptibility [127]. *NOD2* mRNA is undetectable in either colon or small intestine in the CAGE data, supporting the view that it is rapidly down-regulated during monocyte differentiation to macrophages. *NOD2* is believed to be expressed in Paneth cells, and to influence their functions including production of defensins. If *NOD2* is expressed in Paneth cells, it is likely to be at a very low level, since Paneth cell markers such as *DEFA6*, and stem cell markers such as *LGR5*, were easily detected in the total intestine mRNA. Our data suggest that NOD2 is more likely to have a function in monocyte differentiation. Furthermore, we suggest that *NOD2* is not necessarily responsible for the genetic linkage on 16q, since the neighbouring genes, *CYLD* and *SNX20* are tightly co-regulated and lie within the interval of maximal association. The linkage to *NOD2* is seen even in families where there is no coding variation in *NOD2*, and an extended haplotype includes *CYLD* and the shared enhancer/promoter region between *SNX20/NOD2* [128]. Similarly, the case for biological roles of autophagy in Crohn’s disease is also based upon linkage to a coding variant, T300A, in a key gene, *ATG16L1*, but in this case also the link to function has been elusive [129] and the expression and functional data suggest a more likely candidate in the neighbouring *INPPP5D* (SHIP1) gene.

If macrophage differentiation in response to CSF1 is a key event in mucosal macrophage anergy to intestinal flora, *CSF1* itself, the alternate CSF1R ligand, *IL34*, and the receptor *CSF1R*, would also be obvious candidate genes for IBD susceptibility. Two recent studies have focused on elevated *IL34* expression in IBD [130,131]. Both claim that *IL34* is expressed in uninflamed intestinal mucosa, but the FANTOM5 data indicate that the levels are very low. The *IL34* gene has two promoters, one expressed highly in skin and spleen, and the other in brain, consistent with evidence from the mouse *IL34* knockout that *IL34* controls the development of Langerhans cells and microglia [132]. Based upon the data herein, the elevated expression of *IL34* mRNA and protein [130,131] in inflamed mucosa might actually be part of a feedback control to dampen inflammation and initiate repair.

As noted above, *CSF1R* lies within 0.5MB of the *IRGM* locus linked with CD susceptibility by GWA, and might potentially share upstream regulatory elements. Heterozygous mutation in *Csf1r* has been shown to protect against pathology in a colitis model in mice [133]. There is no evidence of association with IBD in the more immediate vicinity of *CSF1R* in the GWA data, but one report based upon direct sequencing in a Acadian American population indicated linkage to an intron 11 SNP [134]. Whole genome sequence data from 1000 genomes and other sources (http://www.ensembl.org), and the recent human exome paper [135] also reveal the existence of numerous likely loss-of-function alleles, with allele frequencies of 1/1000 or more, within the intracellular tyrosine kinase domain of *CSF1R*. So, there may be rare/private mutations in *CSF1R* that impact on IBD susceptibility. *CSF1* was strongly induced in MDM compared to monocytes, suggesting that these cells become autocrine. Variation in the vicinity of an upstream enhancer at the *CSF1* locus is very strongly linked to Paget’s disease [136], and there is some evidence of association with UC at the same interval (S4 Table).

Given the complexity of the transcriptional regulation in monocytes and macrophages, and the lack of robustness discussed above, it is not at all surprising that there are hundreds of ways in which genetic variation can alter the sensitivity to environmental challenge, on the one hand underlying infectious disease resistance, and on the other producing susceptibility to IBD. Some of the genes discussed herein are expressed in both T cells and macrophages. For some, it is clear that expression in macrophages is most relevant to the gut. Macrophage-specific conditional deletion of at least two IBD susceptibility loci, *Il10ra* [137] and *Stat3* knockout [138] has been shown to generate spontaneous colitis in mice. By contrast, in the case of *Ship1 (Inppp5d)*, conditional deletion studies in mice suggest that ileitis present in the constitutive knockout involves both myeloid and T cell dysfunction [101]. Many of the genes implicated in Mendelian very early onset inflammatory bowel diseases (VEOIBD) (reviewed in [139,140]) such as all the components of the phagocyte NADPH oxidase system, are also highly-expressed in blood monocytes. They include some, such as *TTC7A*, *XIAP*, *BTK* and *MEFV* that are down-regulated by CSF1 and therefore meet our criteria. The FANTOM5 data provide a novel insight into some VEOIBD loci. For example, both *STXBP2* and *DOCK8* genes contain distinct monocyte-specific promoters, and the latter also shows evidence of linkage to CD.

Current GWA hits still account for only a small proportion of the heritability in IBD. There are likely to be epistatic interactions between susceptibility loci, which could explain some of the apparently missing heritability [141]. On the other hand, rare coding variants that are specific to particular populations, that might have much larger effect, are invisible to GWA. A recent example is a frame-shift mutation in the *CSFR2B* gene [142,143], also highly-expressed in monocytes and regulated by CSF1 and LPS, associated with Crohn’s disease in Ashkenazi Jews. Several groups have sought evidence of rare protein-coding variation within larger populations, with success limited in large measure by the lack of family-based inheritance data and the lack of a biological hypothesis to prioritise variants [144–146] (reviewed in [6])

Identification of candidate genes within genomic intervals identified by GWA or direct sequencing currently relies on programs such as GRAIL (Gene Relationships Across Implicated Loci). Van Limbergen et al. [8] in a recent review, suggested that knowledge of the genetic architecture of IBD has uncovered biological processes involved in IBD pathogenesis, but that new insights would require knowledge of regulation of transcription of key genes and cell-type specific experiments. Our detailed reanalysis of all of the susceptibility loci thus far identified in GWA support the view that the most relevant cell type to study is the monocyte-macrophage and the prevalent candidate mechanism underlying specific IBD susceptibility is a dysregulation of their differentiation in response to CSF1 and their subsequent response to TLR agonists. Westra et al [61] identified candidate genes based upon an eQTL analysis of blood transcriptional profiles. This is somewhat less sensitive than our approach because monocytes are only a subset of the total blood cells. Nevertheless, supporting our hypothesis, 15/16 separate genes identified as both eQTL and associated with IBD by these authors were also repressed in MDM compared to monocytes and/or induced by LPS in our dataset. Similarly, Fairfax et al. [47] described an eQTL analysis of the response of human monocytes to LPS or IFNγ in which >80% of genes showed genetic association under some condition, and associated a subset of those eQTL to CD susceptibility loci; notably *CARD9 (*which is completely repressed by CSF1). As noted in analysis of the entire FANTOM5 dataset [34,35], SNPs associated with promoters and enhancers are strongly enriched for disease associations. The dataset we have created therefore provides a resource for identification and prioritization of SNPs that are likely to have direct effects on gene expression and causal links to disease.

## Conclusions

This monocyte-macrophage dysregulation hypothesis for IBD is based upon the specific premise that the differentiation of monocytes in response to CSF1, and their subsequent response to LPS, is unique to the gut and the response to the gut microbiome. Clearly, there are also aspects of T cell differentiation that are specific to the gut, for example the Th17 pathway, but there is currently limited direct evidence that any variation in these functions is causally linked to IBD susceptibility. Some of the T cell-associated genes and pathways are controlled by macrophages and their products, including the many cytokines discussed above, so that there is clearly the potential for pleiotropy. In general, variants that affect T cell activation would seem likely to influence multi-system inflammatory disease susceptibility. Similarly, not all variants that affect monocyte differentiation need impact solely on the gut. The obvious role of monocytes in all forms of inflammation could provide an explanation for shared susceptibility loci between IBD and other chronic inflammatory diseases [2], but Fig 7 shows clearly that the CD-specific enrichment is not shared with RA. Our data indicate that the specific association with differentiation in response to CSF1 and/or TLR ligands exemplified by LPS provides an explanation for specific association with inflammation in the unique environment of the GI tract. Of course, the advantage of this hypothesis is that is testable by comparing the transcriptional profiles of MDM from patients and unaffected siblings. That will be a future direction.

## Methods

### Cell culture

A full description of CAGE protocols was provided previously [34]. Ethical approval for the isolation of cells from anonymous donors was granted by the University of Edinburgh Research Ethics Committee. This study was reviewed by the University of Edinburgh College of Medicine Ethics Committee (2009/01) and subsequently renewed by the Lothian Research Ethics Committee (11/AL/0168). Written informed consent was received from all volunteers participating in the study. Blood samples from three donors were used. Peripheral blood mononuclear cells were isolated from a single donation of 320ml of whole blood anticoagulated with acid-citrate-dextrose, by Ficoll density gradient centrifugation. Monocytes were extracted using selection for CD14-positive cells using antibodies on magnetic beads (MACS, Miltenyi Biotech). Monocytes were plated in 6-well plates at 800,000 cells per well in complete media (RPMI containing 10% FCS, 20U/ml penicillin, 20 μg/ml streptomycin, and 2mM l-glutamine (Invitrogen)) with 100ng/ml recombinant human CSF-1 (a gift from Chiron Corp, Emeryville, CA) on tissue culture plastic for 7 days. Supplemental medium (50% of the volume in each well, containing 300ng/ml CSF-1) was added at day 5, and cells were used on day 7. Cells were treated with 10ng/ml bacterial lipopolysaccharide (LPS) from salmonella Minnesota R595 as described previously [21], and then harvested at time points from 15 minutes to 48 hours after treatment by cell lysis, before RNA extraction using the Qiagen RNeasy kit (Qiagen, UK).

### Gene and promoter expression analysis

CAGE library preparation and sequencing was carried out on each RNA sample, and clusters of transcription start sites (CTSS) were identified by decomposition-based peak identification as described [34]. The tag counts associated with CTSS meeting the FANTOM5 ‘robust’ criteria (>200,000 CTSS) were quantified and normalized as described in [34]. As discussed previously, many human genes have multiple promoters, and they are numbered in the FANTOM5 dataset in order of relative abundance of detected CAGE tags. The majority of the analysis herein is based upon quantification of individual promoters. Primary access to all of the FANTOM5 data, including comparable mouse data, is available at http://fantom.gsc.riken.jp/zenbu/

All of the primary data, including the normalized tag counts for each individual replicate at every time point, can be visualized in Table form by accessing the ZENBU browser for the human data directly at: http://fantom.gsc.riken.jp/zenbu/gLyphs/#config=dXO5cTaJBZiiw73fJq2oGD;loc=hg19::chr17:34413143..34438026+

The link opens a specific locus in a genome browser. A search window can be used to move to other loci based upon gene names or genomic interval. One track shows only the LPS time course data. Another shows the entire FANTOM5 data set. The expression of individual promoters can be highlighted and entire set of expression profiles across the primary FANTOM5 data is available for download from the site.

### LPS time course analysis

In order to reduce noise arising from technical variation and biological differences between cells from different volunteers, the expression value for each CTSS was compared to the expression values of time points immediately before or after it, and the corresponding values from the other two donors. Values deviating widely (>3SD) from the mean of this pool of 8 values were replaced with the average of the pool. An average expression value for each CTSS from the three volunteers at each time point was then calculated. CTSS with a minimum expression level of 10 tags per million in at least one timepoint, and with a coefficient of variation > 0.5, were included in expression analysis.

A pairwise coexpression network was generated using the Pearson correlation between expression profiles for each CAGE-defined CTSS across the LPS timecourse. Correlations were included where r ≥ 0.9. Coexpressed clusters were detected using the clustering algorithm, MCL (MCLtools, www.micans.org; inflation value = 1.6). Heatmaps and line graphs of expression data were created using the matplotlib library for Python 2.7.2. For the set of transcription factor genes identified previously [34], total expression associated with all the promoters was aggregated, the three replicates were averaged and only samples that had at least one time point with ≥ 20 tags per million (TPM) were included in the analysis. Gene-based expression data were clustered using BioLayout *Express*^3D^ (http://biolayout.org). The network was again based on correlations of r ≥ 0.9 between genes. A sample-to-sample Pearson correlation for the pattern of transcription factor expression was calculated, and the resultant graph with correlations of r ≥ 0.95 and an MCL inflation value of 2.2 was also displayed using Biolayout Express^3D^

Expression profiles of transcripts of named genes arising near genetic variants significantly associated with Crohn’s disease were examined manually and are described in the text. Following the observation that many of these named transcripts shared a similar pattern of expression with *NOD2*, numerical criteria were determined, to describe the characteristics of this pattern. Subsets of cell types were defined according to the groups in S2 Table. The following criteria were determined *a priori* to describe the expression pattern observed:

- downregulated during monocyte differentiation to macrophage ('unstimulated_monocyte>unstimulated_MDM')
- specifically induced in interferon-stimulated monocytes ('IFN_monocyte>everythingelse')
- specifically induced in monocytes after other inflammatory stimuli ('inflammatory_monocyte>everythingelse')

To assess the association of these candidates with IBD susceptibility, Crohn’s disease summary p-values were obtained from the International Inflammatory Bowel Disease Genetics Consortium (ftp://ftp.sanger.ac.uk/pub4/ibdgenetics/cd-meta.txt.gz.). For comparison, we applied the same criteria to loci putatively associated with ulcerative colitis [ftp://ftp.sanger.ac.uk/pub4/ibdgenetics/ ucmeta-sumstats.txt.gz], rheumatoid arthritis [http://plaza.umin.ac.jp/~yokada/datasource/software.htm] [147] and to a comparable size set of genes associated with unrelated pathologies: the level of LDL cholesterol in blood [http://csg.sph.umich.edu/abecasis/ public/lipids2013/] [148], height [http://portals.broadinstitute.org/collaboration/giant/index.php/GIANT_consortium_data_files] [149]. The ZENBU Browser cited above also shows individual tracks for the p values for association of SNPs with each of the chosen traits, so that they can be precisely aligned with enhancers and promoters within chosen loci.

For each of these studies, GWAS variants with p-values stronger than a permissive threshold (p<1e-6) were counted in 1000 equally-sized bins above and below all TSS in the input set (observed counts), and compared to the absolute count of all variants genotyped in the same study in the same distance bins from all TSS in the input set (expected counts under the null hypothesis that no enrichment exists). For Crohn's disease, ulcerative colitis and the mixed phenotype of inflammatory bowel disease, linkage disequilibrium score regression analyses reveal strong evidence of polygenicity, supporting our decision to draw inferences from variants that do not meet stringent criteria for genome-wide significance. These analyses were conducted by Bulik-Sullivan et al [150] using the same summary p-value data that we use here. The ratio of observed:expected ratio was plotted for each bin (Fig 7). Summary p-values for this set of loci were computed using PASCAL [151] to calculate the total “burden of significance” within a range of +/-100kb from each TSS region, for each GWAS study, and then to quantify the probability of such a signal arising by chance using the PASCAL pathway enrichment feature to compensate for linkage disequilibrium.

## Supporting information

S1 Fig Clusters of coexpressed promoters regulated during the response of monocyte-derived macrophages to LPS. (PNG)

S2 Fig Time course of regulation of selected LPS-inducible promoters and enhancers in their immediate genomic vicinity. (PDF)

S1 Table Gene names and profiles of coexpressed transcription factor clusters described in Fig 1. (XLSX)

S2 Table Names of genes associated with clusters shown in Fig 2. (DOCX)

S3 Table Detailed curation of genomic regions associated with susceptibility to IBD (XLSX)

S4 Table Genes differentially repressed or induced by CSF1, and annotation of association with IBD susceptibility loci. (XLSX)

S5 Table The set of promoters regulated in monocytes by activation stimuli and down-regulated during differentiation. (XLSX)

S6 Table Literature survey of mutations in mice associated with altered responses to LPS in vitro or in vivo. (XLSX)

S7 Table Membership of the FANTOM Consortium. (DOCX)
