# Supplementary figures and images for: Analysis of the human monocyte-derived macrophage transcriptome and response to lipopolysaccharide provides new insights into genetic aetiology of inflammatory bowel disease
Source: PLoS Genet. 2017 Mar 6;13(3):e1006641. doi: 10.1371/journal.pgen.1006641 (PMC5358891; doi:10.1371/journal.pgen.1006641)

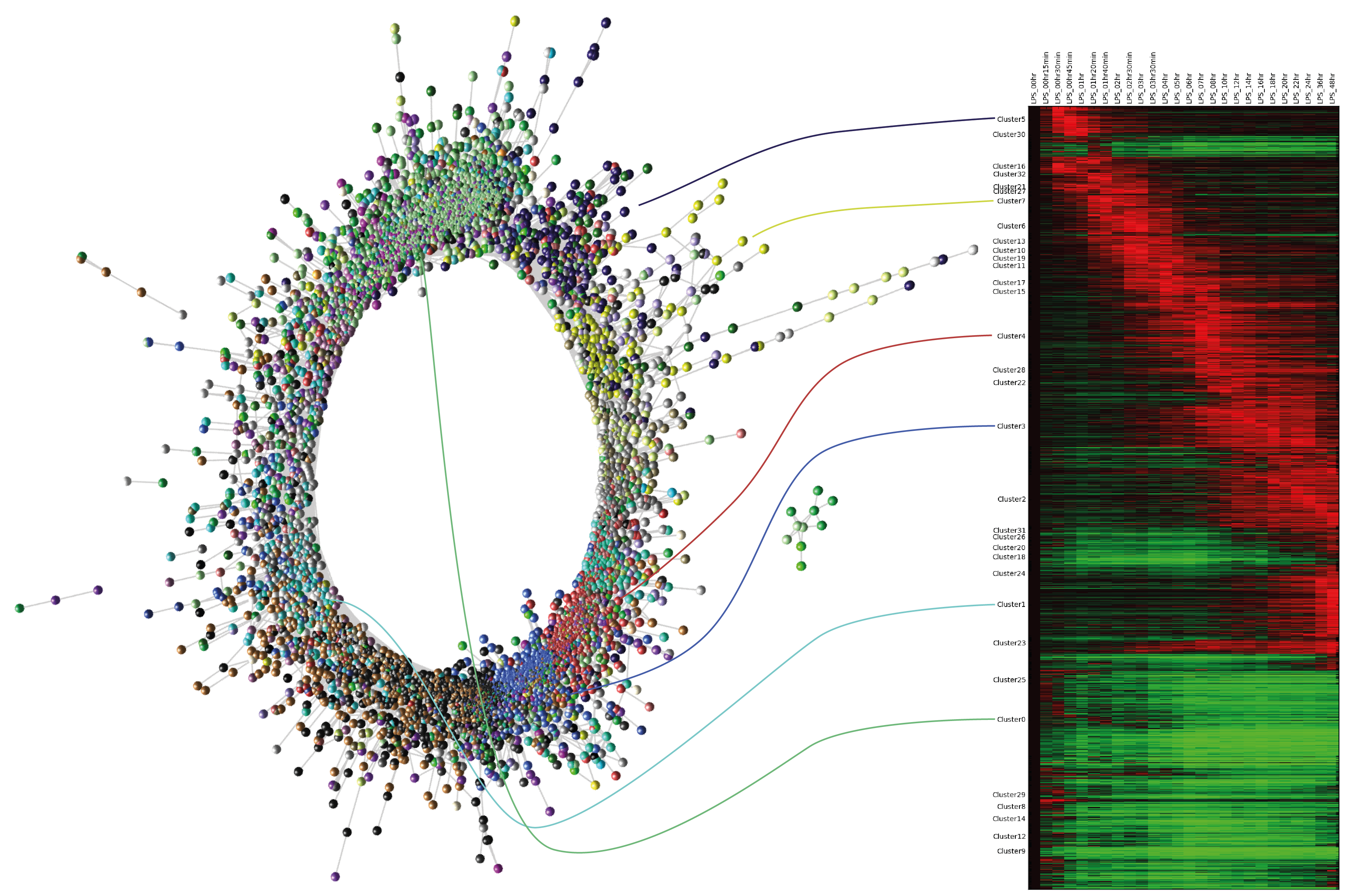

Supplement: S1 Fig — (PNG) [file pgen.1006641.s001.png]

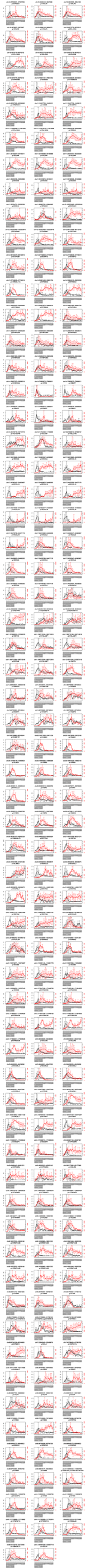

Supplement: S2 Fig — (PDF) [file pgen.1006641.s002.pdf]
