# Supplementary material for: Analysis of the human monocyte-derived macrophage transcriptome and response to lipopolysaccharide provides new insights into genetic aetiology of inflammatory bowel disease
Source: PLoS Genet. 2017 Mar 6;13(3):e1006641. doi: 10.1371/journal.pgen.1006641 (PMC5358891; doi:10.1371/journal.pgen.1006641)
Supplement: S2 Table — (DOCX) [file pgen.1006641.s004.docx]

Supplementary Table 2

Summary of Clusters

Cluster_0

Repressed across time course, maximal by 12 hours.

Includes many cell-cycle-associated genes

A2M, AB384407, AB527524, ABHD6, ABI3, ACCN2, ACE, ACOX2, ACP5, ACSF2, ACSF3, ACSS2, ADAP2, ADCY7, ADK, ADORA3, ADRA2B, AF271776, AFAP1L1, AFTPH, AGPAT9, AHNAK, AIF1, AIG1, AIM1, AK095416, AK097116, AK124456, AKAP11, ALDH3A2, ALDH7A1, ALK, ALOX5AP, AMDHD1, AMICA1, ANGPT4, ANKH, APBA1, APPL2, ARHGAP22, ARHGDIB, ARL5A, ASB1, ASB2, ASRGL1, ASTN2, ATAD2, ATG4C, ATG7, ATP10D, ATP8A1, ATP8B4, ATXN2L, AZI1, B7H6, BC000042, BCAT1, BIN1, BIRC5, BLVRB, BRAT1, BRI3BP, C10orf128, C11orf45, C12orf52, C15orf52, C16orf53, C17orf76-AS1, C19orf35, C1orf112, C1orf187, C20orf123, C20orf27, C20orf96, C21orf56, C7orf58, C8orf55, CA2, CAD, CALM1,p2, CALML4, CAMK2B, CAPN3, CASK, CASP10, CBFA2T3, CC2D2A, CCNA2, CCNB2, CCNY, CCPG1,p3, CD163L1, CD200R1, CD22, CD300LB, CD302, CD36, CD3EAP, CD63, CDC6, CDCA3, CDCA5, CDK1, CDK6, CDKN2C, CEBPA, CENPA, CENPF, CENPK, CENPW, CEP55, CHCHD6, CHST13, CIAO1, CIITA, CKAP2, CLCN5, CLIP4, CMBL, CMTM7, CNNM3, CNRIP1, CNTRL, COLEC12, CORO2A, COTL1, CREB3L2, CRYBB1, CYBRD1, CYSLTR1, DAB2, DBP, DDAH2, DEPTOR, DHDH, DHRS11, DHRS3, DHRS9, DIAPH3, DIXDC1, DNASE2B, DOCK10, DOCK3, DOPEY2, DPEP2, DPY19L3, DVL2, EEF1DP3, EEPD1, ELMO1, EMB, ENC1, ENST00000305812,p1, ENST00000425320, ENST00000429730, ENST00000438482, ENST00000444125, ENST00000448869, ENST00000452793, ENST00000454380, ENST00000455434, ENST00000455981, ENST00000460796, ENST00000471276, ENST00000495324, ENST00000503469, ENST00000529938, ENST00000559487, EPAS1, EVI2B, F13A1, FA2H, FABP4, FABP5, FAIM3, FAM109A, FAM125A, FAM190A, FAM190B, FAM198B, FAM70A, FANCE, FBP1, FBXO38, FES, FGFR1, FGL2, FHL1, FKBP15, FLJ33630, FN3K, FRMD3, FUCA1, GAL3ST4, GALNT12, GAS7, GATM, GFOD1, GGTA1P, GJA5, GLIPR1, GLT8D1, GNG2, GPD1, GPD1L, GPER, GPR34, GREB1, HADH, HAGHL, HAMP, HDLBP, HELLS, HES2, HGF, HIST1H1A, HIST1H2BC,p3, HIST1H2BH, HIST1H2BN, HIST1H3A,p1, HIST1H3A,p10, HIST1H3A,p4, HIST1H4A,p1, HIST1H4A,p3, HIST1H4A,p5, HIST1H4A,p8, HIST1H4C, HOMER3, HPCAL1, HPGD, HPGDS, HS3ST2, HSD11B1L, HSPB1, HTR2B, HTR7, HYI, IFT122, IFT172, IGF1, IL16, IL1R1, IQCK, IQGAP2, IQSEC1, ITGA4, ITGB1BP1, ITGB1BP3, ITPK1, ITPKB, ITSN1, IVNS1ABP, KANK2, KCNAB2, KCNC4, KCNJ5, KIAA0101, KIAA0528, KIAA0930, KIAA1598, KIAA1841, KIAA1919, KIF17, KIF20B, KIF22, KIF2C, KLHL13, KRT79, LAT, LAYN, LDLRAP1, LGALSL, LGI2, LILRA2, LIMD2, LOC100128252, LOC100233209, LOC100505564, LOC100507392, LOC283104, LOC375190, LOC440149, LRP3, LTA4H, LTC4S, LY9, LYPD1, MACF1, MAF, MAP2K6, MARC1, MARVELD1, MAST3, MATK, MAZ, MBNL3, MBOAT1, MBP, MCM4, MCOLN1, ME1, ME3, MEF2C, METTL21A, MEX3B, MGAT5, MIR3654, MLST8, MMD, MPEG1, MRGPRF, MRPL37, MS4A6A, MSR1, MTND4P12, MXD4, MXI1, MYBPH, MYO1D, MYOZ1, NCAPD2, NCAPG, NCAPG2, NCF2, NEDD9, NEK6, NES, NFATC3, NFKBID, NRGN, NRP1, NUMB, NUSAP1, O3FAR1, OLFML3, OSBPL7, OXA1L, P2RX1, P2RY11, PABPC4, PAQR5, PARVG, PAX8, PCMTD2, PDE3B, PDGFC, PDLIM2, PHYHD1, PID1, PIK3IP1, PITHD1, PKD1L1, PLA2G15, PLCB2, PLEC, PLK1, PLXDC2, PNKD, PODXL, PPARG, PPM1L, PPP1R21, PPP3CA, PRADC1, PRKACB, PRKAR1A, PRKCB, PROC, PSTPIP1, PTGFRN, PTPLA, PTPRO, QPRT, RAPH1, RASA1, RASGRP4, RASSF1, RB1, RCBTB2, RDH13, RGL2, RGS19, RGS20, RHOBTB2, RIN2, RIN3, RNASE1, RNASE6, RNF125, RNF128, RNF166, RNF213, RPL34P26, RPS6KA2, RPTOR, RRAGD, RRM2, RTN4, S100A1, S100A13, SAMD1, SAMM50, SCAMP5, SCARB1, SDC1, SDC3, SEPT9, SERINC5, SERTAD2, SGK1, SH2D3C, SH3PXD2A, SHMT1, SIRPB1, SLAMF8, SLC22A18, SLC26A11, SLC2A8, SLC30A3, SLC35B2, SLC36A1, SLC37A2, SLC38A6, SLC39A10, SLC40A1, SLC46A1, SLC6A7, SLC9A7, SLCO2B1, SNHG13, SNX24, SNX5, SORL1, SPARC, SPINT1, SPIRE1, SPOCD1, SPRY1, SPTBN1, SPTBN2, SRGAP3, STAB1, STIP1, STK32C, STX10, SUZ12P, SYS1, TACC1, TACC3, TBC1D10C, TBC1D17, TBC1D4, TBXAS1, TEX264, TFCP2L1, THRA, TIMM8A, TK1, TK2, TLR5, TM7SF4, TMC6, TMC8, TMEM117, TMEM151A, TMEM223, TMEM37, TMEM64, TNFRSF11A, TNFRSF14, TNFRSF21, TNFSF12-TNFSF13,p2, TNFSF13, TNS1, TOP2A, TP53INP1, TPD52L1, TPD52L2, TREM2, TRIM54, TSPAN10, TSPAN15, TTC3, TXNDC16, TYMS, UBE2C, UHRF1, UNC13D, VNN1, VSIG4, WDR11, WDR62, WWP1, XPOT, YIF1A, YPEL4, YWHAZ, ZBED3, ZDHHC14, ZFPM1, ZNF263, ZNF589, ZNF706, ZNF714, ZNF836, ZSWIM7, ZWINT, ZYX, uc003qic.1, uc011mfh.1.

Cluster_1

Peaks at 48 hours

AB528056, AB590576, ADAMDEC1, ADRBK1, AHCYL1, AHR, AKAP13, AKT3, AL832168, ALDH2, ALDOA, ALDOC, AMPD3, AMT, ANO9, ANXA3, AOAH, AP2M1, APBA2, AQP9, ARID5B, ASNS, ASS1, ATG2A, B4GALT1, BAZ2B, BCL6, BCL9L, BST1, BTBD8, C10orf54, C16orf93, C19orf66, C1R, C1RL, C1S, C1orf122, C2,p6, C20orf112, C3, C6orf223, C6orf225, C7orf58, C9orf72, CA12, CAB39, CACNA2D4, CASS4, CCDC115, CCDC163P, CCL15, CCL17, CCL18, CCL23, CCPG1, CCPG1,p1, CD14, CD300E, CD55, CDK19, CDK9, CDKN2D, CEACAM3, CEACAM4, CELF1, CEP19, CEP192, CEP97, CES1, CES1P1, CFB, CFLAR, CHIC1, CHPF, CKAP4, CLDN12, CLEC4D, CLEC4E, CLEC6A, CLU, CP, CR1, CRISPLD2, CSNK1A1, CSTA, CTSH, CXCL16, CXCL5, CXCL6, CYB5R3, CYP2S1, CYTIP, DDX24, DDX5, DEDD2, DGKA, DGKG, DHRS13, DMAP1, DNAJC18, DOCK10, DUSP22, DYSF, EBI3, ENPP2, ENST00000425002, ENST00000428765, ENST00000436469, ENST00000454651, ENST00000498986,p1, ENST00000503539,p1, ENST00000512322, ENST00000522817, ENST00000533341,p3, ENST00000542875, ENST00000546086, EPB41L3, EPOR, ERBB2IP, ETV5, FADS1, FAM124A, FAM168A, FAM198B, FAM20A, FAM55C, FBRS, FBXL4, FBXO10, FBXO5, FCRLA, FILIP1L, FLOT2, FMNL1, FOSL2, FPR1, FPR2, FRAT1, FRMD4A, FTH1P8, FTX, FYN, GAS7, GFM2, GK, GK5, GLIS3, GLT1D1, GPANK1, GPI, GPR65, GRB2, GRINA, GYPC, H1F0, H1FX, H6PD, HAVCR2, HDGFRP3, HLA-F, HMGN1, HNMT, HNRNPH1, IDS, IFI16, IFI6, IFT88, IGFBP7, IL12RB1, IL21R, IL32, ITGAV, JMJD7-PLA2G4B,p1, KANK1, KCNMB4, KIAA0182, KIAA1383, KIAA1432, KIFC3, KLF7, KRT7, LAD1, LAMB3, LBP, LDHA, LGALS2, LHFPL2, LINC00528, LMO4, LNPEP, LOC149837, LOC388813, LOC389634, LPAR1, LRRC61, LTBP4, LYRM1, LYSMD2, MAGED2, MAP1LC3A, MAPKAPK3, MARCH1, MARCO, MATL2963, MBD2, MDK, MDM1, MET, METTL7A, MFSD11, MKNK2, MLLT11, MMP14, MMP2, MSH3, MT1A, MT1B, MT1DP, MT1E, MT1F, MT1G, MT1H, MT1L,p1, MT1M, MT1X, MTX1, MUCL1, MYH11, NBEAL2, NCOA4, NCOA7, NDP, NDRG1, NDST1, NFE2L2, NKG7, NNMT, NR3C1, OLFML2B, OSTF1, OTUD1, P2RY13, PACSIN2, PAF1, PC, PCBP1-AS1, PDCD1LG2, PDE4D, PDE4DIP, PDPN, PDZD7, PHACTR1, PHF12, PHF21A, PIF1, PKD2, PKIB, PLD1, PLEKHA7, PMP22, PNPLA2, PNPLA6, PNRC1, POU2F1, PPAP2B, PPP1R15A, PPP1R1A, PROCR, PTAFR, PTGES, PTPN12, PTPN9, PTPRC, QSOX1, RAB34, RARRES1, RASAL3, RASSF5, RBM33, RERE, RFX2, RG9MTD2, RGS2, RHBDD2, RPGRIP1, RPL13P5,p2, RRAD, RRM2B, RTN2, RUFY3, RUNX2, S100A12, S100A8, S100A9, SAMD10, SCG3, SDR39U1, SEBOX,p2, SEC22B, SELM, SEMA6B, SERPINA1, SERPINB1, SERPINB6, SESN2, SHISA5, SIGLEC10, SIPA1L1, SIRPB2, SLA, SLC11A1, SLC25A37, SLC2A5, SLC30A1, SLC31A2, SLC35E3, SLC39A1, SLC39A8, SLC3A2, SLC7A11, SLC7A7, SLC9A7P1, SMARCD3, SMOX, SMPDL3A, SPATC1, SREBF1, SRGN, STARD13, STX1A, STXBP5, SUPT5H, SYT11, TFDP2, TLR1, TMCC1, TMEM132A, TMEM135, TMEM158, TMEM173, TMEM176A, TMEM176B, TMEM205, TMEM229B, TMEM71, TMEM87A, TNFRSF14, TNFRSF6B, TNFRSF8, TNIK, TNPO2, TOMM6, TRAF3IP3, TREM1, TRIM6, TSEN34, TTBK2, TTYH3, TUBB3, TUBE1, U94903, UBE2D3, UBR1, UGP2, ULK1, UNC13D, USP13, VAMP1, VASN, VCAN, VDR, VEGFA, VILL, VNN2, WAS, WWC3, X07061,p2, ZBTB2, ZER1, ZHX2, ZKSCAN1, ZMYM2, ZMYM6NB, ZNF385A, ZNF484, ZNF655, ZNF747,p2, ZSCAN12, ZSWIM4, uc001zcj.2, uc002ctj.1, uc003whs.1.

Cluster_2

Still rising at 48 hours

A4GALT, AB462940, ACHE, ACSL1, ADAM10, ADAM28, ADAMDEC1, ADC, ADM2, ADNP, AFTPH, AIFM2, AIM1, AIM2, AK125571, ALKBH4, ANKRD1, ANKRD44, ANO8, ANPEP, ANTXR2, APBA3, APBB2, APOBEC3A, APOBEC3H, APOL1, ARHGAP25, ARHGAP30, ARHGEF11, ARID1B, ARID5B, ARL6IP5, ARSB, ASB1, ASB3,p2, ATP1A4, AZI2, B4GALT1, BBS12, BC043279, BCL10, BRMS1L, BTN3A1, BTN3A3, C14orf182, C17orf49, C1QTNF1, C1orf122, C20orf112, C20orf160, C3AR1, C5orf41, C8orf31, C9orf46, CABIN1, CAMK2A, CARM1, CASP7, CBX3, CCDC134, CCDC50, CCL1, CCL15, CCL23, CCND3, CCR1, CD82, CDC26, CDHR3, CDK13, CDKN1B, CDKN2D, CEBPB, CEPT1, CES2, CFB, CFLAR, CHI3L2, CHRNA1, CHST11, CHST2, CKAP4, CLDN12, CLEC2D, CMAS, CRY2, CSF2RB, CTDSP1, CTSL1, CXCL13, CXCL16, CYP1B1, DCAF17, DDI2, DDX11, DDX21, DDX24, DDX60, DNAJC15, DNAJC7, DOCK7, DRD4, DTNB, EBI3, EED, ENPP4, ENST00000309775, ENST00000339867, ENST00000393255, ENST00000417456, ENST00000424989, ENST00000443143, ENST00000444374, ENST00000448344, ENST00000515771,p1, ENST00000558536, EPB41L3, EPM2AIP1, ERLIN1, ERO1L, FAM179B, FAM192A, FAM20A, FAM26F, FAM60A, FAM65B, FCHO2, FILIP1L, FOXN2, FTX, FUT8, FYB, GIMAP2, GIMAP5, GIMAP6, GNA15, GNS, GPANK1, GPX8, GRAMD1A, GRB2, GRINA, GYPC, HAVCR2, HDAC7, HIST2H2AA3,p1, HLA-A, HLA-DPA3, HNRNPUL1, HOMEZ, HSCB, HSD11B1, HTATIP2, IDO1, IFI16, IFI27, IFI6, IFIT3, IFITM1, IFITM2, IFNAR1, IGFBP7, IL10RB, IL21R, IL32, IL4I1, IL7R, IPPK, ITGB8, ITPR3, JAK3, JTB, JUND, JUP, KCTD1, KDM4C, KIAA0247, KIAA1267, KLK10, LAD1, LAMB1, LARP7, LCP1, LDLR, LGALS3BP, LHFPL2, LITAF, LMBR1L, LMO4, LOC100133091, LOC100133669, LOC100507551, LRG1, LRP10, LRRC61, LY6E, LY75-CD302,p1, M69039, MAGEF1, MAP2K6, MARCH1, MCM5, METTL21B, MGA, MKNK2, MLXIP, MMP14, MMP25, MMP3, MMP7, MN1, MT1A,p1, MT1M, MT1P2, MTF1, MUC1, MX1, MYEOV, MYO1G, MYPOP, NCAM1, NDUFB3, NFE2L3, NIPAL2, NKAP, NKAPL, NME7, NSUN7, NXT1, OAS2, OAZ2, OCRL, ODF2L, ODF3B, OSBPL1A, OTUD5, PAQR3, PARP10, PARP15, PARP3, PARVA, PCGF2, PDE1B, PDE4A, PDE4B, PDE9A, PDS5B, PELO, PHF3, PIAS3, PICALM, PILRA, PLAC8, PLD1, PLEKHG2, PLEKHG3, PLP2, PLSCR1, PLTP, PNKD, PNRC1, POLK, PPFIA4, PPP1CB, PPP2R4, PRELID1, PRICKLE3, PRKAG2, PROCR, PRR3, PTGR1, RALBP1, RANBP9, RASSF4, RBBP9, RBPJ, RCC2, RELB, RGL1, RGS19, RHEBL1, RIN1, RPIA, RRAS, RTN4RL2, SCN1B, SEMA4A, SEMA4B, SEMA4D, SERPINA1, SERPING1, SERPINH1, SESN2, SETD5, SH3PXD2B, SHISA5, SIRPA, SLAMF7, SLC11A2, SLC2A6, SLC30A1, SLC39A14, SLC39A8, SLC50A1, SNX10, SNX20, SOAT1, SPAG4, SPECC1, SPHK2, SPPL2A, SPTLC2, SREBF1, SRPK1, SSFA2, SSH1, SSPN, STOM, SUPT3H, SYNPO2, TAGLN2, TBC1D9, TDO2, TGIF1, TIMP1, TLE4, TLR8, TM9SF4, TMEM120A, TMEM171, TMEM173, TMEM176A, TMEM176B, TMEM189-UBE2V1,p1, TMEM216, TNFSF13B, TRAF2, TSC22D3, TSFM, TSPAN3, TSTD2, TTBK2, TTC9C, UBC, UBD, UBE2D3, UCK2, UGP2, ULK2, UNC93B1, USF1, USP33, VDR, WARS, WDR54, XPO1, ZBTB2, ZNF175, ZNF187, ZNF23, ZNF292, ZNF335, ZNF384, ZNF442, ZNF687, uc002hzy.2.

Cluster_3

Induced slow and late, peaks at 12-14 hours

AB464084, ABI1, ACLY, ACSL4, ADAP2, ADAR, ADARB1, AEN, AES, AHCYL2, AIFM2, AIM2, AK092830, AK126179, AK297892, AK4, AKAP2,p5, AKAP2,p7, AKT2, AMPD3, ANO7L1, APOBEC3A, APOL1, APOL3, ARHGAP27, ARHGEF10L, ARHGEF11, ARNTL2, ATF5, ATG3, ATP2C1, ATXN7L1, BCL2L14, BST2, C14orf159, C15orf48, C17orf67, C1orf122, C22orf28, C5orf15, C5orf51, C9orf46, CASP1, CASP3, CCDC50, CCL1, CCL19, CCL7, CCL8, CCR1, CD38, CD40, CD46, CD70, CDC25B, CDC26, CHKB-CPT1B, CHST11, CLEC2D, CMKLR1, CMPK2, CNDP2, CNP, COX4NB, CUL4B, CYP27B1, DCK, DEFB1, DICER1, DNAJA1, DNAJC15, DPP4, DPYSL2, DRAP1, DYNLT1, EBI3, EIF1AY, ENDOD1, ENST00000429685, ENST00000436697,p2, ENST00000447329, ENST00000557195, ERO1L, ETV7, EXTL2, FAM113A, FAM122C, FAM125A, FAM189B, FAM26F, FBXO6, FBXO7, FCAMR, FFAR2, FSTL1, FTSJD2, GADD45G, GANC, GBP1, GBP4, GMPR, GPD2, GRINA, GSDMD, GSN, HERC5, HERC6, HLA-A, HLA-F, HPS5, HSD11B1, HSH2D, HSPA1B, IDO1, IFI35, IFI44L, IFITM1, IFITM2, IFITM3, IGF2BP3, IL15RA, IL18BP, IL1RN, IL2RA, IL4I1, ILK, IRF2, ISG20, ITPA, KAT2B, KIAA0040, KIFAP3, LAG3, LAMP3, LAP3, LDHA, LGALS1, LGALS9, LILRA1, LILRA5, LILRB1, LILRB2, LOC100131733, LRP12, LYSMD2, MAOA, MDFIC, MGAT1, MIA3, MLLT6, MMP12, MNDA, MOV10, MSC, MT1F, MTFP1, MX1, MX2, MYO1G, NARF, NLRC5, NMI, NNT, NT5C3, NTN1, NUB1, OAS1, OAS2, OAS3, ODF2L, OGFRL1, OLIG1, OSBPL9, PARP10, PARP11, PARP14, PARP15, PARP9, PCGF5, PDCD10, PDCD1LG2, PFKP, PGAM1, PHF15, PIK3AP1, PKNOX1, PLA1A, PML, PPA1, PPAN-P2RY11,p3, PPP2R2A, PPP3CC, PSD3, PSMA3, PSMA6, PSMB9, PSME1, PSME2, PSME2P2, PTPN2, PXN, RAB43, RABGAP1L, RARRES3, RASGRP3, RCN1, RDX, RFTN1, RGL1, RGS20, RSAD2, RUSC1, SAMD9, SAP18, SEC24D, SECTM1, SEMA4D, SEPT4, SERPING1, SH3BP2, SIN3A, SLC11A2, SLC31A2, SMU1, SMURF1, SOD2, SP110, SP140, SPATS2L, SPHK1, SRP54, SSB, STAP1, STAT1, STAT2, STBD1, STOML1, SUMO2P3, TAP1, TAP2, TFEC, TIA1, TIAM2, TINF2, TLE4, TMCC2, TMEM110, TMEM140, TMEM171, TMEM219, TMEM63B, TNFRSF14, TNFSF10, TNFSF13B, TOMM5, TOR1AIP1, TOR1B, TRANK1, TRIM38, TRIM5, TTC9C, TXN, UBE2D3, UBE2F, UBE2L6, UBTF, UNC93B1, VAMP5, WARS, WDR45, WIPF1, YEATS2, ZDHHC9, ZNF296, ZNF384, ZNF702P, uc003mmj.1.

Cluster_4

Slow induction, peaks at 6-8 hours

AB464274, ABCA1, ABCC4, ADA, ADORA2A, AEN, AGPAT3, AGPAT5, AHSA1, AKIRIN2, AMPD3, ANGPTL4, ANKIB1, ANKRD33B, APOL2, APOL6, ARHGAP31, ARHGEF10L, ARID3A, ARIH1, ATF5, BAALC, BAHCC1, BASP1, BATF, BATF2, BAZ1A, BBX, BCAR1, BCL3, BMPR2, C19orf66, C1orf21, C2orf76, C5orf56, CASP7, CASP8, CAST, CBX4, CBX6, CCDC109B, CCR7, CD274, CD40, CD97, CDC42SE2, CDKN2A, CERS6, CFLAR, CHD8, CKB, CLIC1, CMPK2, CRELD2, CSK, CTNNBL1, CU677281, CU691854, DEK, DICER1, DLGAP4, DNAJC6, DNPEP, DPYSL2, DTX3L, DYNC1H1, DYRK1A, EDARADD, EDEM1, EIF2S2P4, ELF4, ELMO2, ENST00000419808, ENST00000434052, ENST00000512978, ENST00000518916, EP300, EPSTI1, ERO1LB, ETV7, EXOSC9, FAM123B, FAM125B, FAM129A, FAM177A1, FAM60A, FGD2, FKBP5, FNDC3A, FOXP4, FPGS, FSCN1, FSD1L, FTSJD2, FUT11, FUT4, GBP1, GCH1, GPR180, GTF2E2, GTPBP1, GTPBP2, HAPLN3, HERC5, HERC6, HES4, HESX1, HLX, HNRNPF, HSPA1A,p2, IGFBP4, IL10, IL2RA, ILK, INHBA, ISG15, ISG20, ITGA9, ITGB8, ITPRIPL2, JAK2, KAT2B, KCNS3, KCTD14, KIAA0040, KIAA0226, KIAA1199, KIAA1671, KLF3, KPNB1, LAMP3, LBH, LDLRAD3, LGALS17A, LHFP, LILRB2, LINC00158, LMNB1, LOC154761, LRRFIP1, LSS, LTA, LYN, MACF1, MAFK, MAGI2-AS3, MARCKSL1, MBD1, MBD5, MCOLN2, MED13L, MESDC1, MFN1, MICALL1, MIER1, MX1, MYO10, MYOF, N4BP1, NEDD4L, NKX3-1, NOD1, NOTCH2, NT5C3, NXF1, OAS2, OAS3, OBFC2A, OGFRL1, OPTN, ORAI2, PAF1, PAIP1, PANX1, PARP12, PARP14, PDCD1LG2, PDGFRL, PEX26,p2, PFKFB3, PFN1P12, PHACTR2, PHRF1, PI4K2B, PLAGL1, PLEKHO1, PML, POLR3D, POU2F2, PPM1K, PRIC285, PRKCH, PRLR, PRPF3, PRPF4B, PTPN2, PWP1, RAB7L1, RAP2C, RBCK1, RBM6, RET, RFTN1, RIPK2, RTF1, SAMD9, SEC24A, SETX, SH3BP2, SLAMF7, SLC15A3, SLC16A7, SLC25A28, SLC37A1, SLC41A1, SLC41A2, SLC43A3, SLCO4A1, SMG7, SNHG15, SNX27, SNX7, SOD2, SPATS2, SPTLC2, SSTR2, ST3GAL4, STAMBPL1, STAT3, STAT5A, STAU1, STBD1, TANK, TGFA, TJP1, TMCC2, TMEM110-MUSTN1,p1, TMEM63B, TMX1, TNFRSF10A, TNFRSF1B, TNFRSF4, TNFSF10, TOP1, TPM4, TRIM14, TRIM22, TRIM25, TSC22D2, UBE2S, USP14, USP18, USP25, USP28, VAV2, VDAC1, WARS, XRN1, ZEB2, ZNF107, ZNF277, ZNF513, ZNRF2.

Cluster_5

Early-response genes, peak at 45-60 minutes

AJ227912, AJ227917, AK024925, AK075122, ATG7, CHD2, CU691365, CXCL2, DUSP1, DUSP2, EGR1, EGR2, EGR3, ENST00000318816, ENST00000363009, ENST00000365207, ENST00000405359, ENST00000423112, FOS, FRMD4B, IKZF1, JUN, KIF23, KLF2, LILRB5, LOC152225, LOC646329, LOC730227, LRP5L, MEX3C, MIR3648, NFIL3, NFKBIZ, NR2F6, NR4A1, NTHL1, PRDM1, PSTPIP1, RNU12, S69623, SGK1, SLC48A1, TSNARE1, VPS18, uc003lyl.3.

Cluster_6
Early-response genes, peak at 150-180mins

ADAM17, ARAP2, ATF7IP2, ATXN7L1, BC007549, BC009749, C2CD4B, CCDC82, CCL20, CCL3L1,p1, CCRL2, CD69, CDK4, CH25H, DENND4A, DUSP6, ENST00000427998,p1, ENST00000517983, ENST00000543494, GADD45A, GPR183, HCAR2, HCAR3, HIAT1, IL10, IL1B, IL6, IL8, IRAK2, KLF4, KMO, LCP2, MAMLD1, MIR4741, MSC, NAV1, NEU4, NFKBIZ, PDSS1, PELI1, PHLDB1, PLEK, PPP1R15B, PTGS2, REM1, SEPP1, SGMS2, SGPP2, TAOK3, TBK1, THAP2, TNFAIP8, TNFSF18, TNFSF9, TNIP2, TSLP, UBAC2, UPP1, XBP1, ZBTB38.

Cluster_7

Early-response genes, peak at 120-140mins

ABL2, ACTN2, ACVR2A, AK092443, ARMC5, ATP2C1, BC033346, BCL2L1, CCRL2, CDKN2A, CXCL1, DRAM1, ENST00000435434, F3, GEM, GPR183, GTF2F2, IL1B, JAG1, LINC00346, LOC100302650, MAFF, MIR155, MYC, NFATC1, NFKBIZ, OTUD1, PDGFB, PLEKHF2, PMAIP1, REPIN1, RHOB, SPRED2, TNF, ZBTB10, ZFP36L2.

Cluster_8

Fully-repressed after 2-3 hours

ACSS1, ADNP, ADRBK1, AMN1, APOC1, BBS5, BHLHE41, BICD1, BRCA1, C5orf13, CD14, CEP78, CHN2, CNST, CPOX, CREB5, ENST00000366185,p1, ENST00000426200,p1, ENST00000457535, ENST00000508745,p1, ENST00000527986, FAM13A, FAM173B, FAM53B, FAM78A, FBXO5, FRMD4B, GAS2L3, GIT2, GNB1L,p2, GNB5, GRAMD4, HHEX, HIST1H3A,p6, ING2, INPPL1, KBTBD6, LOC100506023, LOC283143, LPAR6, LYL1, MAN2A2, MGAT1, NR1D2, OTUB2, PCM1, PDXK, PIK3C2B, PLEC, RAB3IL1, RABGEF1, RCBTB2, RIN3, RNF166, RPP25, SETDB2, SLMO1, SNAI3, SOWAHC, SOWAHD, SPIN2B, STARD13, STT3B, TBC1D12, TCFL5, TGFBR1, TRIM32, UBL4A, USP2, WFS1, WHSC1, ZEB2, ZHX3, ZNF331, ZNF362, ZNF383, ZNF610, ZNF93.

Cluster_9
Fully-repressed after 4-5 hours

ABHD15, ACP6, ANLN, ARHGEF3, BCOR, BDNF-AS1, C20orf197, C8orf55, CCDC112, CCND2, CD180, CDT1, CECR6, CIITA, CKAP2L, CLN8, CMTM8, DAB2, DHRS3, DIRAS1, DLGAP5, ENST00000409910, ENST00000411579,p1, ENST00000425254, ENST00000451152, ENST00000514288, ENST00000559298, EVI2B, EYA2, FAM13A, FGD4, FMN1, FRMD4B, FUT10, FYB, GAS2L3, GCOM1,p1, GPR82, HDAC9, HGF, LDHD, LGR4, LOC151174, MAD2L1, MAF, MARK2, MBP, MS4A4E, MTUS1, MXD3, NAA16, PLCG1, RAB32, RABGAP1L, RARA, RASGRP3, RGS12, RGS18, RIN3, RREB1, SLC36A1, SLC7A8, SNAI3, SOCS6, SOX12, SPC25, SSBP3, TACC2, TBC1D4, TLR7, TMEM150A, TNFSF14, UNG, ZNF703.

Cluster_10

Induced at 150-180mins, slow decline

AK026758, ARAP2, BCL2A1, BIRC2, BIRC3, C11orf96, CCL3, CCL4, CD86, CFLAR, CXCL10, ELF1, ENST00000433442, ENST00000447307, ENST00000518417, HDX, IFI44, IFIT2, IFIT3, IFIT5, IL6, M55024, MAP3K8, NUMA1, OSR2, PLEK, RBM47, RIN2, RIPK2, S81242, SERPINB2, SPHK1, TMEM63B, TNFAIP8, TOR1AIP1, ZBTB38, ZBTB43, ZC3HAV1.

Cluster_11

Induced at 180-210 mins, slow decline

AB384300, AKAP2, ALCAM, ATP2B1, BCL2, CCNJ, CSRNP1, DSTYK, DYRK3, EHD1, ELOVL7, ENST00000447307, GPR132, IFIH1, IL1RAP, IL23A, LITAF, LRCH1, MACF1, MAP4K4, MAPKAPK2, MMP19, MSC, NBN, NFKB1, NINJ1, NIPAL4, NR4A3, P2RX7, PIM1, PRR5, PTK2B, RAP2C, RASSF5, RGS16, RIMKLB, SAV1, SERPINB9, SOCS1, SPSB1, SRC, STAT5A, STK40, TIFA, TNFSF15, TRIP10, TUBB2A, XBP1.

Cluster_12

Repressed across time course, but reinduced late

ABHD2, ABLIM3, AGBL5, ARHGAP4, BCKDHA,p2, C1orf162, CD14, CDA, CHD4, CPEB1, CTNNBIP1, CXCL16, DUSP7, ELMO1, EMR3, ENST00000419300, EVI2A, EVI2B, FGFR1, HDAC5, HLTF, HPS4, HS2ST1, LACC1, LTA4H, LTB4R, LYPLAL1, MAML3, MTSS1, NBEAL2, NEDD9, NT5DC2, PLXDC2, PYGL, RAB11FIP1, RASSF2, SEPT9, SLC2A4RG, SLC30A3, SLC46A3, SLC47A1, SLC4A7, SLCO2B1, SNX29, SNX5, SPIRE1, SYPL2, TBC1D2, TBXAS1, TGFBI, TGFBR2, TK2, TM6SF1, TPCN1, TRPV4, UBE4B, UBTD1, VASH1, ZNF219.

Cluster_15

Induced, peaks at 4-5 hours

ABTB2, AMOTL2, ARHGAP22, ARHGAP23, BATF, BATF3, BCAR1, BIRC2, BOC, C12orf23, C14orf43, C3orf38, C3orf52, C4orf46, CEBPD, CNKSR3, DYRK1A, ELF4, ENST00000516198, EXT1, GET4, GFPT2, GREM1, HUWE1, IL15, IL23A, IL27, KIAA1109, LCOR, LRRC32, MAP3K5, MFHAS1, NANS, NBL1, NFIX, PDSS1, POLR3D, PRDM8, PRPF3, RAB21, RELA, RHOU, RIN2, SCN9A, SLAMF7, SMG7, SNAI1, SPATA13, TANC1, TAP1, TCEA1, TMEM2, TRIM25, UBXN2A, ZNF710, ZNRF2, uc003iwy.1, uc003jnb.1.

Cluster_17

Induced, peaks at 3-4 hours

ALCAM, ARID5A, BCL6, CCDC117, CFLAR, CKB, CXCL9, CYTH1, DDX58, DHX58, DUSP5, ELOVL7, ENST00000510284,p1, FNDC3B, GBP5, GPBP1, IFIH1, IL15, IRF4, IRG1, KIAA0368, MASTL, MB21D1, N4BP2L1, NCOA7, NFKB2, OASL, PPM1K, RAB33A, RELA, RIPK1, RNF138, SLC7A5, SOCS3, STX11, TFPI2, TMEM39A, TNFAIP2, TNFRSF9, TRIM36, TRIP10.

Cluster 28

Induced by 6-7hrs, remained elevated

CD44, CDC42EP2, CDYL2, DENND5A, FSD1L, GRAMD1A, HS3ST3B1, IL10, IL15, IL7R, ITGB8, KDM6A, MCOLN2, MSC, P2RY6, PLSCR1, PPP3CC, PSMA6, SBNO2, SLC2A6, SOD2, SP100, SPHK1, TLE4, TNFAIP3, TNIP1, TRAF3IP2, TYMP, XAF1.

Cluster 42

Induced, peaks at 80-100 mins

ENST00000454380, ERRFI1, IFNB1, ITPK1, MAP3K4, MECP2, PLEKHF2, PPP1R15A, TMEM39A, TNFAIP3, TRAF1, ZBTB10.

Cluster 30

ACVR2A, AIF1, CXCL3, EGR4, ENST00000536099, PPP1R15A, SESTD1.
